# Global Coinfections with Bacteria, Fungi, and Respiratory Viruses in Children with SARS-CoV-2: A Systematic Review and Meta-Analysis

**DOI:** 10.3390/tropicalmed7110380

**Published:** 2022-11-15

**Authors:** Saad Alhumaid, Muneera Alabdulqader, Nourah Al Dossary, Zainab Al Alawi, Abdulrahman A. Alnaim, Koblan M. Al Mutared, Khalid Al Noaim, Mohammed A. Al Ghamdi, Suha Jafar Albahrani, Abdulaziz A. Alahmari, Sarah Mahmoud Al Hajji Mohammed, Yameen Ali Almatawah, Omar Musa Bayameen, Ahmed Abdulwhab Alismaeel, Sherifah Khaled Alzamil, Samiah Ahmad Alturki, Zahra’a Radi Albrahim, Nasreen Ahmad Al Bagshi, Hesham Yousef Alshawareb, Jaafar Abdullah Alhudar, Qassim Abdulatif Algurairy, Samirah Mansour Alghadeer, Hassan Ali Alhadab, Taleb Nasser Aljubran, Yousif Ahmad Alabdulaly, Abbas Al Mutair, Ali A. Rabaan

**Affiliations:** 1 Administration of Pharmaceutical Care, Al-Ahsa Health Cluster, Ministry of Health, Al-Ahsa 31982, Saudi Arabia; 2 Pediatric Nephrology Specialty, Pediatric Department, Medical College, King Faisal University, Al-Ahsa 31982, Saudi Arabia; 3 General Surgery Department, Alomran General Hospital, Ministry of Health, Al-Ahsa 36358, Saudi Arabia; 4 Division of Allergy and Immunology, College of Medicine, King Faisal University, Al-Ahsa 31982, Saudi Arabia; 5 Department of Pediatrics, College of Medicine, King Faisal University, Al-Ahsa 31982, Saudi Arabia; 6 Administration of Pharmaceutical Care, Ministry of Health, Najran 66255, Saudi Arabia; 7 Department of Pediatrics, King Fahad Hospital of the University, College of Medicine, Imam Abdulrahman Bin Faisal University, Dammam 34212, Saudi Arabia; 8 Division of Diabetology, Family Medicine Department, College of Medicine, King Faisal University, Al-Ahsa 36364, Saudi Arabia; 9 Pharmacy Department, Prince Saud Bin Jalawi Hospital, Al-Ahsa 36424, Saudi Arabia; 10 Division of Infectious Diseases and Infection Control, Pediatric Department, Maternity and Children Hospital, Ministry of Health, Al-Ahsa 36422, Saudi Arabia; 11 Public Health Administration, Directorate of Health Affairs, Ministry of Health, Al-Ahsa 36441, Saudi Arabia; 12 Southern Sector, Primary Care Medicine, Al-Ahsa Health Cluster, Ministry of Health, Al-Ahsa 36421, Saudi Arabia; 13 Regional Medical Supply, Al-Ahsa Health Cluster, Ministry of Health, Al-Ahsa 36361, Saudi Arabia; 14 Nutrition Department, King Fahad Hofuf Hospital, Ministry of Health, Al-Ahsa 36441, Saudi Arabia; 15 Infection Prevention and Control Administration, Al-Ahsa Health Cluster, Ministry of Health, Al-Ahsa 36421, Saudi Arabia; 16 Ambulatory Transportation Administration, Al-Ahsa Health Cluster, Ministry of Health, Al-Ahsa 36421, Saudi Arabia; 17 Quality Assurance and Patient Safety Administration, Directorate of Health Affairs, Ministry of Health, Al-Ahsa 36441, Saudi Arabia; 18 Research Center, Almoosa Specialist Hospital, Al-Ahsa 36342, Saudi Arabia; 19 College of Nursing, Princess Norah Bint Abdulrahman University, Riyadh 11564, Saudi Arabia; 20 School of Nursing, Wollongong University, Wollongong, NSW 2522, Australia; 21 Department of Nursing, Prince Sultan Military College, Dhahran 34313, Saudi Arabia; 22 Molecular Diagnostic Laboratory, Johns Hopkins Aramco Healthcare, Dhahran 31311, Saudi Arabia; 23 College of Medicine, Alfaisal University, Riyadh 11533, Saudi Arabia; 24 Department of Public Health/Nutrition, The University of Haripur, Haripur 22620, Khyber Pakhtunkhwa, Pakistan

**Keywords:** bacterial, children, co-infection, coinfection, concurrent, COVID-19, fungal, meta-analysis, pediatric, SARS-CoV-2, viral, systematic review

## Abstract

Background: Coinfection with bacteria, fungi, and respiratory viruses has been described as a factor associated with more severe clinical outcomes in children with COVID-19. Such coinfections in children with COVID-19 have been reported to increase morbidity and mortality. Objectives: To identify the type and proportion of coinfections with SARS-CoV-2 and bacteria, fungi, and/or respiratory viruses, and investigate the severity of COVID-19 in children. Methods: For this systematic review and meta-analysis, we searched ProQuest, Medline, Embase, PubMed, CINAHL, Wiley online library, Scopus, and Nature through the Preferred Reporting Items for Systematic Reviews and Meta-Analyses (PRISMA) guidelines for studies on the incidence of COVID-19 in children with bacterial, fungal, and/or respiratory coinfections, published from 1 December 2019 to 1 October 2022, with English language restriction. Results: Of the 169 papers that were identified, 130 articles were included in the systematic review (57 cohort, 52 case report, and 21 case series studies) and 34 articles (23 cohort, eight case series, and three case report studies) were included in the meta-analysis. Of the 17,588 COVID-19 children who were tested for co-pathogens, bacterial, fungal, and/or respiratory viral coinfections were reported (*n* = 1633, 9.3%). The median patient age ranged from 1.4 months to 144 months across studies. There was an increased male predominance in pediatric COVID-19 patients diagnosed with bacterial, fungal, and/or viral coinfections in most of the studies (male gender: *n* = 204, 59.1% compared to female gender: *n* = 141, 40.9%). The majority of the cases belonged to White (Caucasian) (*n* = 441, 53.3%), Asian (*n* = 205, 24.8%), Indian (*n* = 71, 8.6%), and Black (*n* = 51, 6.2%) ethnicities. The overall pooled proportions of children with laboratory-confirmed COVID-19 who had bacterial, fungal, and respiratory viral coinfections were 4.73% (95% CI 3.86 to 5.60, *n* = 445, 34 studies, *I*^2^ 85%, *p* < 0.01), 0.98% (95% CI 0.13 to 1.83, *n* = 17, six studies, *I*^2^ 49%, *p* < 0.08), and 5.41% (95% CI 4.48 to 6.34, *n* = 441, 32 studies, *I*^2^ 87%, *p* < 0.01), respectively. Children with COVID-19 in the ICU had higher coinfections compared to ICU and non-ICU patients, as follows: respiratory viral (6.61%, 95% CI 5.06–8.17, *I*^2^ = 0% versus 5.31%, 95% CI 4.31–6.30, *I*^2^ = 88%) and fungal (1.72%, 95% CI 0.45–2.99, *I*^2^ = 0% versus 0.62%, 95% CI 0.00–1.55, *I*^2^ = 54%); however, COVID-19 children admitted to the ICU had a lower bacterial coinfection compared to the COVID-19 children in the ICU and non-ICU group (3.02%, 95% CI 1.70–4.34, *I*^2^ = 0% versus 4.91%, 95% CI 3.97–5.84, *I*^2^ = 87%). The most common identified virus and bacterium in children with COVID-19 were RSV (*n* = 342, 31.4%) and *Mycoplasma pneumonia* (*n* = 120, 23.1%). Conclusion: Children with COVID-19 seem to have distinctly lower rates of bacterial, fungal, and/or respiratory viral coinfections than adults. RSV and *Mycoplasma pneumonia* were the most common identified virus and bacterium in children infected with SARS-CoV-2. Knowledge of bacterial, fungal, and/or respiratory viral confections has potential diagnostic and treatment implications in COVID-19 children.

## 1. Introduction

**Figure 1:**
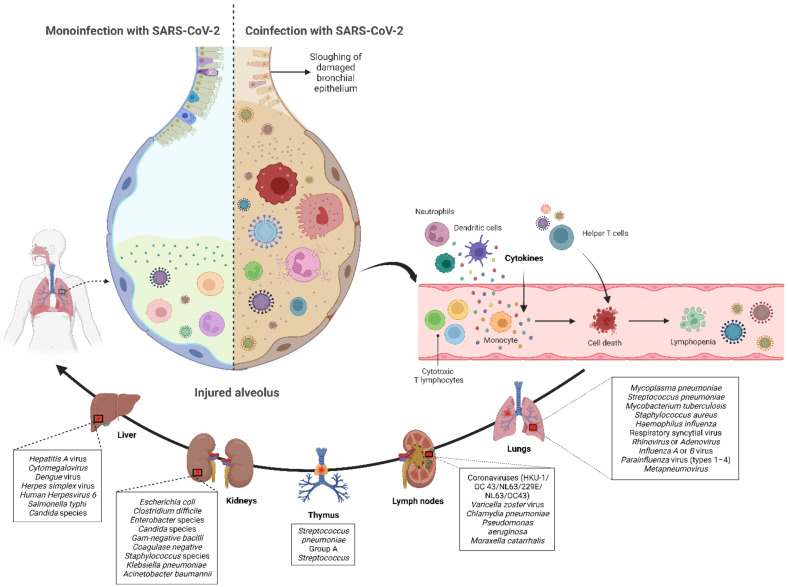
Monoinfection with SARS-CoV-2 results in less severe form of COVID-19 and better prognosis. In contrast, SARS-CoV-2 coinfection with bacteria, fungi, and/or respiratory viruses may intensify the severity of COVID-19 and increase the expression of macrophages, T and B defensive cells that may cause the elevation of inflammatory cytokines such as tumor necrosis factor-alpha, interleukin-1, and interleukin-6 in the infected organs, leading to a hyperinflammatory response by recruiting immune cells.

Although most cases of coronavirus disease 2019 (COVID-19) in pediatric populations are mild or asymptomatic [1], the clinical spectrum of severe acute respiratory syndrome coronavirus 2 (SARS-CoV-2) infection in children ranges from asymptomatic to life-threatening [2,3]. Similar to adults, coinfection with bacteria, fungi, and respiratory viruses has been described as a factor associated with more severe clinical outcomes in children with COVID-19 [4,5,6,7,8,9,10,11]. Such coinfections have been reported to increase morbidity and mortality, therefore, knowledge of bacterial, fungal, and/or respiratory viral confections has potential diagnostic and treatment implications in children infected with SARS-CoV-2. Many studies have shown that COVID-19 children may develop severe diseases, requiring intensive care admission and/or mechanical ventilation because patients rapidly develop acute respiratory distress syndrome and sepsis, leading to death from multiple organ failure [12,13,14,15,16,17,18,19,20,21,22,23]. SARS-CoV-2 is hypothesized to weaken the bodies of children to bacterial, fungal, and/or respiratory viral coinfections [24], yet the mechanism of coinfection has not been fully established, but represents a threat to the respiratory epithelium favoring bacteremia, fungaemia, and/or viraemia (see Figure 1).

There is a lack of systematic reviews and meta-analyses on the type and frequency of coinfection by bacterial, fungal, and/or respiratory viral infections and associated clinical outcomes among COVID-19 children. We aimed to identify the type and proportion of coinfections with SARS-CoV-2 and bacteria, fungi, and/or respiratory viruses, and investigate the severity of COVID-19 in these patients.

## 2. Methods

### 2.1. Design

We followed the Preferred Reporting Items for Systematic Reviews and Meta-Analyses guidelines (PRISMA) in conducting this systematic review and meta-analysis [25]. The following electronic databases were searched: PROQUEST, MEDLINE, EMBASE, PUBMED, CINAHL, WILEY ONLINE LIBRARY, SCOPUS, and NATURE with Full Text. We used the following keywords: (“*COVID-19*” OR “*SARS-CoV-2*” OR “*Severe acute Respiratory Syndrome Coronavirus 2*” OR “*Coronavirus Disease 2019*” OR “*2019 novel coronavirus*”) AND (“*children*” OR “*child*” OR “*paediatric*” OR “*pediatric*” OR “*infant*” OR “*toddler*” OR “*adolescent*” OR “*newborn*”) AND (“*coinfection*” OR “*co-infection*” OR “*cocirculation*” OR “*co-circulation*” OR “*coinfected*” OR “*co-infected*” OR “*co-circulated*” OR “*mixed*” OR “*concurrent*” OR “*concomitant*”). The search was limited to papers published in English between 1 December 2019 and 1 October 2022. Based on the title and abstract of each selected article, we selected those discussing and reporting the occurrence of bacterial, fungal, and/or respiratory viral coinfection in children with COVID-19.

### 2.2. Inclusion–Exclusion Criteria

Inclusion criteria were as follows: (1) published case reports, case series, and cohort studies that focused on children infected with SARS-CoV-2 and bacteria, fungi, and/or respiratory viruses; (2) studies of experimental or observational design reporting the incidence of SARS-CoV-2 infection in pediatric patients with other co-pathogens; (3) language restricted to English. The exclusion criteria were as follows: (1) editorials, commentaries, case and animal studies, reviews, and meta-analyses; (2) studies that did not report data on COVID-19 in coinfected patients; (3) studies that never reported details on identified coinfected cases with SARS-CoV-2 infection; (4) studies that reported coinfection in adult COVID-19 patients; (5) studies that reported coinfection in patients with negative SARS-CoV-2 polymerase chain reaction (PCR) tests; (6) duplicate publications.

### 2.3. Data Extraction

Six authors (Saad Alhumaid, Muneera Alabdulqader, Nourah Al Dossary, Zainab Al Alawi, Abdulrahman A. Alnaim, and Koblan M. Al mutared) critically reviewed all of the studies retrieved and selected those judged to be the most relevant. Data were carefully extracted from the relevant research studies independently. Articles were categorized as case report, case series, or cohort studies. The following data were extracted from selected studies: authors; publication year; study location; study design and setting; number of SARS-CoV-2 children tested for co-pathogens; number of coinfected children; age; proportion of male children; patient ethnicity; number of children with bacterial, fungal, and/or respiratory viral coinfections; total organisms identified; antimicrobials prescribed; laboratory techniques for co-pathogen detection; number of children admitted to intensive care unit (ICU), placed on mechanical ventilation, and/or suffered acute respiratory distress syndrome (ARDS); assessment of study risk of bias; and final treatment outcome (survived or died). These data are noted in Table 1.

### 2.4. Quality Assessment

For many selected cohort studies, the Newcastle–Ottawa scale (NOS) was used to assess the risk of bias, a tool which measures quality in the three parameters of selection, comparability, and exposure/outcome, and allocates a maximum of 4, 2, and 3 points, respectively [26]. High-quality studies are scored greater than 7 on this scale, and moderate-quality studies between 5 and 7 [26]. Otherwise, quality assessment of the selected case report and case series studies was undertaken based on the modified NOS [27]. Items related to the comparability and adjustment were removed from the NOS, and items which focused on selection and representativeness of cases, and the ascertainment of outcomes and exposure, were kept [27]. Modified NOS consists of five items, each of which requires a yes or no response to indicate whether bias is likely, and these items were applied to single-arm studies [27]. Quality of the study was considered good if all five criteria were met, moderate when four were met, and poor when three or less were met. Quality assessment was performed by six authors (Khalid Al Noaim, Mohammed A. Al Ghamdi, Suha Jafar Albahrani, Abdulaziz A. Alahmari, Sarah Mahmoud Al HajjiMohammed, and Yameen Ali Almatawah) independently, with any disagreement to be resolved by consensus.

### 2.5. Data Analysis

The proportion of confirmed COVID-19 children with bacterial, fungal, and/or respiratory viral coinfection were examined. This proportion was further classified based on initial presentation or during the course of the illness. A random effects DerSimonian–Laird model was used, which produces wider confidence intervals (Cis) than a fixed effect model [28]. Results are illustrated using a forest plot. The Cochran’s chi-square (*χ*^2^) and the *I*^2^ statistic provided the tools for examining statistical heterogeneity [29]. An *I*^2^ value of >50% suggested significant heterogeneity [30]. To lower the source of heterogeneity, we conducted a subgroup analysis based on children’s admission to the ICU. To estimate publication bias, funnel plots and Egger’s correlation were used, and a *p*-value < 0.05 was considered to indicate statistical significance. All *p*-values were based on two-sided tests and significance was set at a *p*-value less than 0.05. R version 4.1.0 with the packages *finalfit* and *forestplot* was used for all statistical analyses. Figure 1 was created with BioRender.com (agreement no. NX24IV1VNB) (accessed on 14 October 2022).

## 3. Results

### 3.1. Study Characteristics and Quality

**Figure 2:**
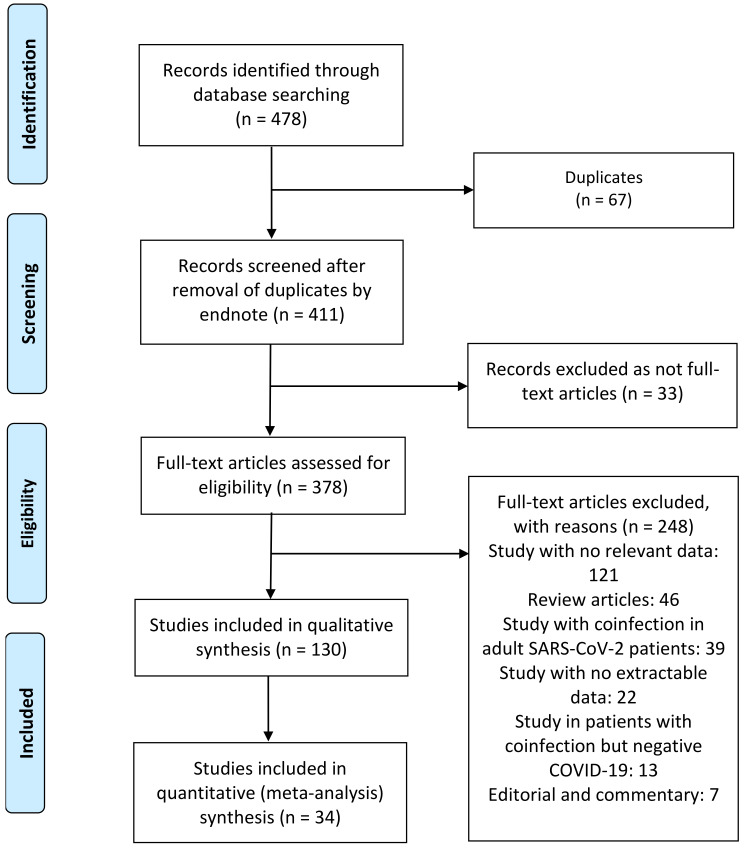
Flow diagram of literature search and data extraction from studies included in the systematic review and meta-analysis.

A total of 130 publications were identified (Figure 2). After scanning titles and abstracts, 67 duplicate articles were discarded. Another 33 irrelevant articles were excluded based on the titles and abstracts. The full texts of the 378 remaining articles were reviewed, and 248 irrelevant articles were excluded. As a result, we identified 130 studies that met our inclusion criteria and reported SARS-CoV-2 infection in pediatric patients with bacterial, fungal, and viral coinfection [4,5,6,7,8,9,10,11,12,13,14,15,16,17,18,19,20,21,22,23,31,32,33,34,35,36,37,38,39,40,41,42,43,44,45,46,47,48,49,50,51,52,53,54,55,56,57,58,59,60,61,62,63,64,65,66,67,68,69,70,71,72,73,74,75,76,77,78,79,80,81,82,83,84,85,86,87,88,89,90,91,92,93,94,95,96,97,98,99,100,101,102,103,104,105,106,107,108,109,110,111,112,113,114,115,116,117,118,119,120,121,122,123,124,125,126,127,128,129,130,131,132,133,134,135,136,137,138,139,140]. The detailed characteristics of the included studies are shown in Table 1. Among these, two articles were preprint versions [64,89]. There were 57 cohort [4,5,6,7,8,9,10,11,12,17,31,32,34,35,36,37,39,41,42,44,49,53,58,66,69,71,73,78,79,80,81,82,83,84,89,90,95,98,99,102,105,108,109,115,116,118,119,123,125,127,129,131,133,134,137,138,139], 52 case report [13,14,15,16,18,19,21,22,23,33,38,40,43,45,46,48,50,51,52,55,56,59,64,65,67,68,70,72,74,75,76,77,86,87,91,93,94,97,104,106,107,110,111,113,114,121,122,124,126,128,130,140], and 21 case series [20,47,54,57,60,61,62,63,85,88,92,96,100,101,103,112,117,120,132,135,136] studies. These studies were conducted in the United States (*n* = 23), China (*n* = 21), India (*n* = 9), Italy (*n* = 7), Iran (*n* = 7), France (*n* = 6), Turkey (*n* = 6), Spain (*n* = 5), Mexico (*n* = 4), Brazil (*n* = 4), Indonesia (*n* = 4), South Africa (*n* = 3), Switzerland (*n* = 3), Poland (*n* = 3), United Kingdom (*n* = 3), Argentina (*n* = 2), Saudi Arabia (*n* = 2), United Arab Emirates (*n* = 1), Portugal (*n* = 1), Malaysia (*n* = 1), Thailand (*n* = 1), Bulgaria (*n* = 1), The Netherlands (*n* = 1), Germany (*n* = 1), Lebanon (*n* = 1), Botswana (*n* = 1), Denmark (*n* = 1), Russia (*n* = 1), Pakistan (*n* = 1), Bangladesh (*n* = 1), Japan (*n* = 1), Greece (*n* = 1), and Canada (*n* = 1). Only four studies were conducted within multiple countries (*n* = 4) [53,109,118,127]. The majority of the studies were single-center [4,6,11,12,13,14,15,16,18,19,21,22,23,32,33,34,35,38,39,40,41,43,44,45,46,48,49,50,51,52,55,56,57,59,60,61,62,64,65,66,67,68,69,70,72,73,74,75,76,77,78,79,80,81,82,83,85,86,87,88,89,90,91,92,93,94,97,98,99,100,101,102,103,104,105,106,107,111,112,113,114,115,116,117,120,121,122,124,126,128,130,132,133,134,138,139,140] and only 33 studies were multicenter [5,7,8,9,10,17,20,31,36,37,42,47,53,54,58,63,71,84,95,96,108,109,110,118,119,123,125,127,129,131,135,136,137]. In some studies, concurrent infection of SARS-CoV-2 with other bacterial, fungal, and/or viral pathogens was investigated in pediatric and adult patients as the population of interest (19/130, 14.6%) [10,17,31,37,47,54,57,62,71,79,82,90,96,102,108,112,118,123,139]. The majority (*n* = 128) of the studies included any hospitalized patient, except for two studies that investigated potential of SARS-CoV-2 transmission in a cluster and genomic analysis of SARS-CoV-2 in a family [47,62], and two studies included only critically ill COVID-19 patients [9,18]. Eleven, four, and one studies exclusively reported on respiratory viral [10,18,31,35,61,71,73,89,101,125,140], bacterial [11,96,100,112], and fungal [90] coinfections, respectively; the remaining 114 studies reported on bacterial, fungal, and respiratory viral coinfections [4,5,6,7,8,9,12,13,14,15,16,17,19,20,21,22,23,32,33,34,36,37,38,39,40,41,42,43,44,45,46,47,48,49,50,51,52,53,54,55,56,57,58,59,60,62,63,64,65,66,67,68,69,70,72,74,75,76,77,78,79,80,81,82,83,84,85,86,87,88,91,92,93,94,95,97,98,99,102,103,104,105,106,107,108,109,110,111,113,114,115,116,117,118,119,120,121,122,123,124,126,127,128,129,130,131,132,133,134,135,136,137,138,139]. Few studies investigated the existence of COVID-19 with *influenza* virus type A and B only [31,101,140], *Mycobacterium tuberculosis* only [11,96,112], respiratory syncytial virus (RSV) only [35,61], *Rhinovirus* only [10,125], *pneumovirus* only [18], *herpes simplex* virus only [71], human coronavirus OC43 only [73], *adenovirus* only [89], *Mycoplasma pneumonia* only [100], and *Candida* species only [90]. Laboratory techniques for co-pathogen detection within studies included 52 that used real-time reverse transcription–polymerase chain reaction (RT-PCR) tests for multiple respiratory viruses [9,10,17,18,31,34,37,41,43,44,46,47,48,49,61,65,66,68,69,71,73,76,79,80,81,82,83,89,91,92,95,99,101,102,110,113,115,116,117,119,123,125,127,129,130,131,132,135,136,137,138,139], 23 that used antibody tests (immunoglobulins M and/or G) [5,8,22,23,45,52,54,56,70,72,77,78,81,85,98,100,104,108,111,120,122,133,140], 42 that used cultures (blood, urine, cerebrospinal fluid, tracheal, nasal discharge, pharyngeal swabs, wound, respiratory secretions, bronchoalveolar lavage, alveolar fluid, sputum, and pleural fluid) [5,6,11,12,13,15,16,20,21,23,38,40,42,50,51,53,55,60,63,64,74,75,84,85,87,88,90,93,96,97,105,106,107,109,112,114,118,121,124,126,128,134], 29 that used two or more laboratory methods (RT-PCR, antibody tests, and/or culture) [4,5,6,7,8,12,20,23,36,39,42,50,52,53,57,58,63,72,77,78,81,84,85,98,105,109,124,126,133], and two that did not specify their testing method [32,33]. Among the 130 included studies, 57 cohort studies were assessed using the NOS: 52 studies were found to be moderate-quality studies (i.e., NOS scores were between 5 and 7) and five studies demonstrated a relatively high quality (i.e., NOS scores > 7). All case reports and case series studies were assessed for bias using the modified NOS. Forty-nine studies were deemed to have high methodological quality, and three exhibited moderate methodological quality; Table 1.

**Table 1:** Summary of the characteristics of the included studies with evidence on SARS-CoV-2 and bacterial, fungal, and/or respiratory viral coinfections in children (*n* = 130), 2020–2022.

Author, Year, Study Location	Study Design, Setting	Number of SARS-CoV-2 Patients Tested for Co-Pathogens, n	Coinfected Patients, n	Age (Months) ^a^	Male, n (%) AND Ethnicity, n ^b^	Bacterial Coinfection, n	Fungal Coinfection, n	Respiratory Viral Coinfection, n	Total Organisms, n	Antimicrobials Used, n	Laboratory Techniques for Co-Pathogen Detection	Admitted to ICU, n	Mechanical Ventilation, n	ARDS, n	Assessment of Study Risk of Bias (Tool Used, Finding) and Treatment Outcome
Aggarwal et al. 2022 [31], India	Retrospective cohort, multicenter	770	4	12, 18, 96, and 72	3 (75) AND 4 Indian	0	0	6	3 *Influenza* A virus 3 *Influenza* B virus	0	RT-PCR for respiratory specimens (viruses) ^c^	0	0	0	(NOS, 7) 4 survived
Al Mansoori et al. 2021 [32], United Arab Emirates	Retrospective cohort, single-center	17	7	Median (IQR), 84 (0–192)	Gender (not reported) AND Ethnicity (not reported)	2	0	5	3 *Rhinovirus* 2 Group A *Streptococcus* 1 *Enterovirus* 1 *Adenovirus*	7 Not reported	RT-PCR for respiratory specimens (viruses) ^c^ Not reported (Group A *Streptococcus*)	0	0	0	(NOS, 6) Treatment outcome (not reported)
Allen-Manzur et al. 2020 [33], Mexico	Retrospective case report, single-center	1	1	6	0 (0) AND 1 Hispanic	1	0	0	1 *Mycobacterium bovis*	1 Not reported	RT-PCR for respiratory specimens (viruses) ^c^ Not reported (*Mycobacterium bovis*)	0	0	0	(Modified NOS, moderate) 1 survived
Alrayes et al. 2022 [34], United States	Retrospective cohort, single-center	13	13	Age group 0–2: 270 (71.3%) patients (RSV coinfection)	Gender (not reported) AND Ethnicity (not reported)	0	0	15	13 RSV 1 *Rhinovirus* 1 *Adenovirus*	13 Not reported	RT-PCR for respiratory specimens (viruses) ^c^	0	0	0	(NOS, 7) 13 survived
Alvares 2021 [35], Brazil	Retrospective cohort, single-center	32	6	Median (IQR), 6	2 (33.3) AND 6 Hispanic	0	0	6	6 RSV	1 Not reported	Chemiluminescence for RSV	1	1	1 Not reported	(NOS, 6) 6 survived
Anderson et al. 2021 [4], United States	Retrospective cohort, single-center	29	10	Age group 168 (42–198): 10 (34.4%) patients Age group 192 (168–204): 9 (31%) patients Age group 102 (72–168): 10 (34.4%) patients	Gender (not reported) AND Ethnicity (not reported)	5	0	6	2 *Staphylococcus aureus* 2 *Escherichia coli* 1 *Salmonella enteritis* 1 *Enterovirus* 1 *Adenovirus* 2 *Rhinovirus* 1 *Parainfluenza* virus 1 EBV	10 Not reported	RT-PCR for respiratory specimens (viruses) ^c^ PCR assays (bacteria)	7	2	3	(NOS, 8) 7 survived 3 died
Andina-Martinez et al. 2022 [36], Spain	Prospective cohort, multicenter	9	2	1.3 and 1.8	1 (50) AND 2 White (Caucasian)	1	0	1	1 *Bordetella pertussis* 1 *Metapneumovirus*	2 Azithromycin	RT-PCR for respiratory specimens (viruses) ^c^ PCR assays (*Mycoplasma pneumoniae*, *Chlamydia pneumoniae* and *Bordetella pertussis*)	1	1	2 Not reported	(NOS, 7) 2 survived
Aragón-Nogales et al. 2022 [12], Mexico	Prospective cohort, single-center	181	2	12 and 24	0 (0) AND 2 Hispanic	1	0	1	1 *Pseudomonas aeruginosa* 1 EBV	1 Cefotaxime 1 Ceftriaxone	RT-PCR for respiratory specimens (viruses) ^c^ Blood culture (bacteria)	2	2	2	(NOS, 7) 2 died
Arguni et al. 2022 [37], Indonesia	Retrospective cohort, multicenter	125	59	Two patients: <12 months to <60 months Six patients: <60 months to <216 months	Gender (not reported) AND 8 Asian	0	0	59	32 *Influenza* A virus 10 *Adenovirus* 16 *Influenza* B virus 1 *Metapneumovirus*	59 Not reported	RT-PCR for respiratory specimens (viruses) ^c^	59 Not reported	59 Not reported	59 Not reported	(NOS, 6) Treatment outcome (not reported)
Arslan et al. 2021 [38], Turkey	Retrospective case report, single-center	1	1	10	1 (100) AND 1 White (Caucasian)	1	0	0	1 MSSA	1 Clindamycin 1 Ceftriaxone	Blood culture (bacteria)	0	0	0	(Modified NOS, high) 1 survived
Aykac et al. 2021 [39], Turkey	Retrospective cohort, single-center	115	37	Median (IQR), 48 (12–132)	Gender (not reported) AND 37 White (Caucasian)	37	0	4	37 *Streptococcus pneumoniae* 2 *Bocavirus* 1 *Rhinovirus* 1 *Parechovirus*	7 Ceftriaxone 7 Azithromycin 7 Ampicillin/sulbactam	RT-PCR for respiratory specimens (viruses) ^c^ PCR assays (*Streptococcus pneumoniae*)	1	1	1	(NOS, 6) Treatment outcome (not reported)
Ayoubzadeh et al. 2021 [40], Canada	Retrospective case report, single-center	1	1	168	1 (100) AND 1 Pakistani	1	0	0	1 Gram-negative *bacilli* 1 *Salmonella Typhi*	1 Meropenem 1 Ampicillin 1 Amoxicillin	Blood culture (bacteria)	0	0	0	(Modified NOS, high) 1 survived
Berksoy et al. 2021 [41], Turkey	Retrospective cohort, single-center	128	21	1 patient: 5 Other patients: not reported	Gender (not reported) AND 21 White (Caucasian)	0	0	23	9 *Rhinovirus* 5 *Metapneumovirus* 4 RSV 3 *Adenovirus* 2 *Bocavirus*	21 Not reported	RT-PCR for respiratory specimens (viruses) ^c^	21 Not reported	0	21 Not reported	(NOS, 6) Treatment outcome (not reported)
Blázquez-Gamero et al. 2021 [42], Spain	Retrospective cohort, multicenter	27	2	1 and 3	Gender (not reported) AND 27 White (Caucasian)	3	0	0	1 *Streptococcus mitis* 1 *Escherichia coli* 1 *Enterobacter cloacae*	2 Ampicillin 1 Gentamycin 1 3rd -generation cephalosporin	RT-PCR for respiratory specimens (viruses) ^c^ Blood culture (bacteria) Urine culture (bacteria)	1	1	1	(NOS, 7) 2 survived
Borocco et al. 2021 [43], France	Retrospective case report, single-center	1	1	156	0 (0) AND 1 Arab	0	0	1	1 EBV	0	RT-PCR for respiratory specimens (viruses) ^c^	0	0	0	(Modified NOS, high) 1 survived
Brothers et al. 2021 [13], United States	Retrospective case report, single-center	1	1	144	0 (0) AND 1 White (Caucasian)	1	1	0	1 MSSA 1 *Candida glabrata*	1 Clindamycin 1 Vancomycin 1 Cefepime 1 Fluconazole 1 Micafungin	Tracheal culture (bacteria) Urine culture (urine)	1	1	1	(Modified NOS, high) 1 died
Cason et al. 2022 [44], Italy	Retrospective cohort, single-center	64	17	Age group <24 was the most frequent)	Gender (not reported) AND 17 White (Caucasian)	0	0	19	1 Other coronaviruses (229E, NL63, and OC43) 12 *Rhinovirus* 4 *Bocavirus* 2 *Adenovirus*	17 Not reported	RT-PCR for respiratory specimens (viruses) ^c^	17 Not reported	17 Not reported	17 Not reported	(NOS, 6) Treatment outcome (not reported)
Chacón-Cruz et al. 2022 [14], Mexico	Retrospective case report, single-center	1	1	84	1 (100) AND 1 Hispanic	1	0	0	1 *Neisseria meningitidis*	1 Amoxicillin 1 Ceftriaxone 1 Doxycycline	PCR assays (*Neisseria meningitidis*)	1 Not reported	1 Not reported	1 Not reported	(Modified NOS, high) 1 died
Chen et al. 2020 [45], China	Retrospective case report, single-center	1	1	144	1 (100) AND 1 Asian	2	0	0	1 *Mycoplasma pneumonia* 1 *Chlamydia pneumoniae*	1 Mezlocillin 1 Ceftizoxime 1 Amoxicillin/clavulanic acid	Serum antibody tests (IgM, IgG)	0	0	1	(Modified NOS, high) 1 survived
Choudhary et al. 2022 [5], United States	Retrospective cohort, multicenter	947	235	Age group <60: 101 (33.9%) patients (viral coinfection) Age group <60: 50 (16.8%) patients (bacterial coinfection)	Gender (not reported) AND Ethnicity (not reported)	123	7	113	75 RSV 113 Viral 123 Bacterial 7 Fungal	123 Antibiotics	RT-PCR for respiratory specimens (viruses) ^c^ Blood culture (bacteria) Serum antibody tests (IgM, IgG)	33	14	235 Not reported	(NOS, 8) 233 survived 2 died
Ciuca et al. 2021 [46], Italy	Retrospective case report, single-center	1	1	72	1 (100) AND 1 Black	0	0	1	1 *Parvovirus* B19	1 Antibiotics	PCR assays (*Parvovirus* B19)	1	1	1	(Modified NOS, high) 1 survived
Danis et al. 2020 [47], France	Retrospective case series, multicenter	12	1	108	1 (100) AND 1 White (Caucasian)	0	0	2	1 *Influenza* A virus 1 *Rhinovirus*	0	RT-PCR for respiratory specimens (viruses) ^c^	0	0	0	(NOS, 7) 1 survived
Danley and Kent 2020 [48], United States	Retrospective case report, single-center	1	1	4	1 (100) AND 1 White (Caucasian)	0	0	1	1 *Adenovirus*	0	RT-PCR for respiratory specimens (viruses) ^c^	0	0	1	(Modified NOS, high) 1 survived
DeBiasi et al. 2020 [49], United States	Retrospective cohort, single-center	63	4	Median, 115.2	Gender (not reported) AND Ethnicity (not reported)	0	0	5	2 *Rhinovirus* 2 RSV 1 Other coronaviruses (229E, NL63, and OC43)	4 Not reported	RT-PCR for respiratory specimens (viruses) ^c^	4 Not reported	4 Not reported	4 Not reported	(NOS, 6) Treatment outcome (not reported)
Demirkan and Yavuz 2021 [50], Turkey	Retrospective case reports, single-center	2	2	84 and 156	0 (0) AND 2 White (Caucasian)	0	2	0	2 Fungal bezoars	1 Meropenem 2 Fluconazole	RT-PCR for respiratory specimens (viruses) ^c^ Blood culture (bacteria)	0	0	0	(Modified NOS, high) 2 survived
Dhanawade et al. 2021 [51], India	Retrospective case report, single-center	1	1	48	0 (0) AND 1 Indian	1	0	0	1 *Mycobacterium tuberculosis*	1 Ceftriaxone 1 Antibiotics 1 Isoniazid 1 Rifampicin 1 Pyrazinamide 1 Ethionamide	CSF culture (bacteria)	1	1	1	(Modified NOS, high) 1 survived
Di Nora et al. 2022 [52], Italy	Retrospective case report, single-center	1	1	24	1 (100) AND 1 White (Caucasian)	0	0	1	1 Human *Herpesvirus* 6	1 Acyclovir 1 Ceftriaxone	CSF PCR assays (viruses) Serum antibody test (IgM)	0	0	0	(Modified NOS, high) 1 survived
Dikranian et al. 2022 [53], Multi-country	Retrospective cohort, multicenter	922	31	Age group ≤6: 136/820 (16.6%) Age group >120 to 180: 182/820 (22.2%) Age group >180 to 216: 189/820 (23%)	Gender (not reported) AND Ethnicity (not reported)	0	0	30	10 *Rhinovirus* 5 RSV 2 *Adenovirus* 1 Coronavirus NL63 1 *Parainfluenza*-2 1 *Parainfluenza*-3 1 *Parainfluenza*-4 1 *Metapneumovirus* 8 Unspecified viruses	31 Not reported	RT-PCR for respiratory specimens (viruses) ^c^ Blood culture (bacteria) Sputum (bacteria)	22	31 Not reported	31 Not reported	(NOS, 6) Treatment outcome (not reported)
Diorio et al. 2020 [6], United States	Prospective cohort, single-center	24	7	Median (IQR), 60 (30–192)	5 (71.4) AND 3 White (Caucasian) 1 Hispanic 3 Black	4	0	5	1 *Parainfluenza* 3 1 *Parainfluenza* 4 2 *Escherichia coli* 1 *Enterovirus* 1 *Adenovirus* 1 *Rhinovirus* 1 MRSA 1 *Salmonella typhi*	7 Not reported	RT-PCR for respiratory specimens (viruses) ^c^ Blood culture (bacteria) Urine (culture)	1	1	1	(NOS, 8) 6 survived 1 died
Dong et al. 2020 [54], China	Retrospective case series, multicenter	11	1	28	1 (100) AND 1 Asian	0	0	1	1 *Cytomegalovirus*	0	Serum antibody test (IgM)	0	0	0	(Modified NOS, high) 1 survived
Essajee et al. 2020 [55], South Africa	Retrospective case report, single-center	1	1	31	0 (0) AND 1 Black	1	0	0	1 *Mycobacterium tuberculosis*	1 Antibiotics 1 Isoniazid 1 Rifampicin 1 Pyrazinamide 1 Ethionamide	Blood culture (bacteria)	0	0	1	(Modified NOS, high) 1 survived
Ferdous et al. 2021 [56], Bangladesh	Retrospective case report, single-center	1	1	96	0 (0) AND 1 Bangladeshi	0	0	1	1 *Dengue* virus	1 Antibiotics	*Dengue* NS1 antigen	1	1	1	(Modified NOS, high) 1 survived
Freij et al. 2020 [15], United States	Retrospective case report, single-center	1	1	60	0 (0) AND 1 Black	2	0	0	1 *Mycobacterium tuberculosis* 1 Group A *Streptococcus*	1 Amoxicillin 1 Azithromycin	CSF culture (bacteria)	1	1	0	(Modified NOS, high) 1 died
Frost et al. 2022 [57], United States	Retrospective case series, single-center	7	6	Median (IQR), 16 (7–30)	5 (83.3) AND 5 Hispanic	14	0	5	1 *Adenovirus* 1 *Metapneumovirus* 2 *Rhinovirus* 1 *Enterovirus* 4 *Streptococcus pneumoniae* 5 *Haemophilus influenza* 3 *Moraxella catarrhalis* 2 *Staphylococcus aureus*	7 Not reported	RT-PCR for respiratory specimens (viruses) ^c^ PCR assays (bacteria)	0	0	0	(Modified NOS, high) 6 survived
Garazzino et al. 2021 [7], Italy	Retrospective cohort, multicenter	515	69	Median (IQR), 87 (17–149)	Gender (not reported) AND 69 White (Caucasian)	32	0	45	45 Unspecified viruses 32 Unspecified bacteria	69 Not reported	RT-PCR for respiratory specimens (viruses) ^c^ PCR assays (bacteria)	3	3	2	(NOS, 7) 67 survived 2 died
Garazzino et al. 2020 [58], Italy	Retrospective cohort, multicenter	168	10	Median (IQR), 28 (4–115)	Gender (not reported) AND 10 White (Caucasian)	1	0	10	3 RSV 3 *Rhinovirus* 2 EBV 1 *Influenza* A virus 1 Other coronaviruses (229E, NL63, and OC43) 1 *Streptococcus pneumoniae*	10 Not reported	RT-PCR for respiratory specimens (viruses) ^c^ PCR assays (bacteria)	2	2	2	(NOS, 6) Treatment outcome (not reported)
Goussard et al. 2020 [59], South Africa	Retrospective case report, single-center	1	1	29	1 (100)AND 1 Black	1	0	0	1 Rifampicin-sensitive *Mycobacterium tuberculosis*	1 Antibiotics 1 Isoniazid 1 Rifampicin 1 Pyrazinamide 1 Ethionamide 1 Amoxicillin/clavulanic acid	PCR assay for gastric aspirate (*Mycobacterium tuberculosis*)	0	0	0	(Modified NOS, high) 1 survived
Guy et al. 2022 [60], United States	Retrospective case series, single-center	6	6	Median (IQR), 144 (42–168)	5 (83.3) AND 5 Black 1 White (Caucasian)	5	0	0	1 *Streptococcus intermedius* 2 *Prevotella* species 2 *Streptococcus constellatus*	4 Ceftriaxone 3 Clindamycin 2 Amoxicillin/clavulanic acid 1 Penicillin 2 Metronidazole 1 Ampicillin/sulbactam 2 Vancomycin 1 Cefdinir	Nasal discharge (culture)	0	0	0	(Modified NOS, high) 6 survived
Halabi et al. 2022 [61], United States	Retrospective and prospective case series, single-center	18	18	Median (IQR), 6 (2–36)	11 (61.1) AND Ethnicity (not reported)	0	0	22	18 RSV 3 *Rhinovirus* 1 *Parainfluenza* virus	18 Not reported	RT-PCR for respiratory specimens (viruses) ^c^	9	2	2	(Modified NOS, high) Treatment outcome (not reported)
Hamzavi et al. 2020 [16], Iran	Retrospective case report, single-center	1	1	168	1 (100) AND 1 Persian	1	0	0	1 *Staphylococcus aureus*	1 Vancomycin 1 Meropenem	Blood (culture)	1	1	1	(Modified NOS, high) 1 died
Hare et al. 2021 [62], United Kingdom	Retrospective case series, single-center	7	1	22	0 (0) AND 1 White (Caucasian)	0	0	1	1 *Rhinovirus*	0	RT-PCR for respiratory specimens (viruses) ^c^	0	0	0	(Modified NOS, high) 1 survived
Hashemi et al. 2021 (17], Iran	Retrospective cohort, multicenter	105	5	Age group 0 to 168: 5 (4.8%) patients (viral coinfection)	4 (80) AND 5 Persian	0	0	5	3 *Metapneumovirus* 1 *Bocavirus* 1 *Influenza* A virus	5 Not reported	RT-PCR for respiratory specimens (viruses) ^c^	5	5	5	(NOS, 7) 5 died
Hashemi et al. 2021 [18], Iran	Retrospective case reports, single-center	3	3	13, 72, and 72	2 (66.6) AND 3 Persian	0	0	3	3 *Metapneumovirus*	3 Not reported	RT-PCR for respiratory specimens (viruses) ^c^	3	3	3	(Modified NOS, high) 3 died
Hassoun et al. 2021 [63], United States	Retrospective case series, multicenter	8	6	Median (IQR), 1.4 (0.5–1.6)	5 (83.3) AND 2 Black 2 White (Caucasian) 1 Hispanic 1 Indian	1	0	6	5 RSV 1 *Rhinovirus* 1 *Escherichia coli*	6 Antibiotics	RT-PCR for respiratory specimens (viruses) ^c^ Urine (culture)	0	0	0	(Modified NOS, high) 6 survived
He et al. 2020 [8], China	Retrospective cohort, multicenter	15	4	Median (IQR), 72 (36–84)	3 (75) AND 4 Asian	2	2	0	2 Unspecified bacteria 2 Unspecified fungi	4 Antibiotics	RT-PCR for respiratory specimens (viruses) ^c^ Sputum (bacteria) G assay and GM assay (fungi) Serum antibody test (IgM)	2	2	2	(NOS, 7) 2 survived 2 died
Hertzberg et al. 2020 [64], United States	Retrospective case reports, single-center	3	3	2, 24 and 60	2 (66.7) AND Ethnicity (not reported)	1	0	2	2 *Rhinovirus* 1 *Bordetella pertussis*	1 Azithromycin	RT-PCR for respiratory specimens (viruses) ^c^ Blood (culture)	1	0	0	(Modified NOS, moderate) 3 survived
Jarmoliński et al. 2021 [65], Poland	Retrospective case report, single-center	1	1	108	0 (0) AND 1 White (Caucasian)	0	0	2	1 *Metapneumovirus* 1 RSV	1 Piperacillin/tazobactam 1 Amikacin 1 Azithromycin 1 Cefepime 1 Micafungin 1 Acyclovir	RT-PCR for respiratory specimens (viruses) ^c^	0	0	0	(Modified NOS, high) 1 survived
Jiang et al. 2020 [66], China	Retrospective cohort, single-center	161	2	80 and 42	0 (0) AND 2 Asian	1	0	3	1 RSV 2 *Metapneumovirus* 1 *Mycoplasma pneumonia*	2 Antibiotics	RT-PCR for respiratory specimens (viruses) ^c^	1	0	1	(NOS, 7) 2 survived
Jose et al. 2021 [67], Mexico	Retrospective case report, single-center	1	1	84	1 (100) AND 1 Hispanic	0	0	1	1 *Dengue* virus	1 Amoxicillin 1 Trimethoprim/sulfamethoxazole 1 Clindamycin 1 3rd -generation cephalosporin 1 Ceftriaxone 1 Acyclovir	RT-PCR for respiratory specimens (viruses) ^c^ DENV RTqPCR (*dengue*)	1	1	1	(Modified NOS, high) 1 survived
Kakuya et al. 2020 [68], Japan	Retrospective case report, single-center	3	2	132 and 60	2 (100) AND 2 Asian	0	0	2	1 *Influenza* A virus 1 *Metapneumovirus*	1 Ceftriaxone	RT-PCR for respiratory specimens (viruses) ^c^	0	0	0	(Modified NOS, high) 2 survived
Kanthimathinathan et al. 2021 [9], United Kingdom	Retrospective cohort, multicenter	73	17	Median (IQR), 120 (12–156)	Gender (not reported) AND 6 White (Caucasian) 5 Asian 4 Black	6	4	14	3 *Pseudomonas aeruginosa* 2 *Klebsiella pneumoniae* 1 *Acinetobacter baumannii* 2 *Adenovirus* 2 *Influenza* 2 *Parainfluenza* 2 *Rhinovirus* 1 *Metapneumovirus* 1 RSV 4 *Cytomegalovirus* 4 Unspecified fungi	3 Amoxicillin/clavulanic acid 1 Azithromycin 2 Clarithromycin 1 Piperacillin/tazobactam 1 Gentamicin	RT-PCR for respiratory specimens (viruses) ^c^	17	7	10	(NOS, 8) 16 survived 1 died
Karaaslan et al. 2021 [69], Turkey	Retrospective cohort, single-center	93	7	Mean ± SD, 10.99 ± 6.44	5 (71.4) AND 7 White (Caucasian)	1	0	7	2 *Rhinovirus* 2 Coronavirus NL63 1 *Adenovirus* 1 *Mycoplasma pneumoniae* 1 *Rhinovirus* 1 *Adenovirus*	7 Antibiotics	RT-PCR for respiratory specimens (viruses) ^c^	0	0	0	(NOS, 7) 7 survived
Karimi et al. 2020 [70], Iran	Retrospective case report, single-center	1	1	144	1 (100) AND 1 Persian	0	0	1	1 *Varicella zoster* virus	1 Azithromycin	Serum antibody tests (IgM and IgG)	0	0	0	(Modified NOS, high) 1 survived
Katz et al. 2022 [71], United States	Retrospective cohort, multicenter	16	2	72 and 120	1 (50) AND 2 White (Caucasian)	0	0	2	2 *Herpes simplex* virus	2 Not reported	RT-PCR for respiratory specimens (viruses) ^c^	2 Not reported	2 Not reported	2 Not reported	(NOS, 6) Treatment outcome (not reported)
Kazi et al. 2021 [72], India	Retrospective case report, single-center	1	1	9	0 (0) AND 1 Indian	0	0	1	1 *Dengue* virus	1 Ceftriaxone 1 Vancomycin 1 Doxycycline	RT-PCR for respiratory specimens (viruses) ^c^ DENV RTqPCR (*dengue*) IgM antibody test from CSF (dengue)	1	1	1	(Modified NOS, high) 1 survived
Keshavarz Valian et al. 2022 [73], Iran	Retrospective cohort, single-center	25	2	Mean ± SD, 58.8 ± 51.6	Gender (not reported) AND 2 Persian	0	0	2	2 Human coronavirus OC43	2 Not reported	RT-PCR for respiratory specimens (viruses) ^c^	2 Not reported	2 Not reported	2 Not reported	(NOS, 6) Treatment outcome (not reported)
Khataniar et al. 2022 [74], India	Retrospective case report, single-center	1	1	168	1 (100) AND 1 Indian	1	0	0	1 *Mycobacterium tuberculosis*	1 Meropenem 1 Vancomycin 1 Ceftriaxone 1 Amikacin 1 Levofloxacin 1 Isoniazid 1 Rifampicin 1 Pyrazinamide 1 Ethionamide	CSF culture (bacteria)	1	1	1	(Modified NOS, high) 1 survived
Lambrou et al. 2022 [75], Greece	Retrospective case report, single-center	1	1	36	0 (0) AND 1 White (Caucasian)	1	0	0	1 *Escherichia hermannii*	1 Piperacillin/tazobactam 1 Amikacin 1 Teicoplanin 1 Meropenem 1 Micafungin	Blood (culture)	0	0	0	(Modified NOS, high) 1 survived
Le Glass et al. 2021 [10], France	Retrospective cohort, multicenter	2159	58	Age group <180: 25 (43.1%) patients (rhinovirus coinfection)	33 (56.9) AND Ethnicity (not reported)	58 Not reported	58 Not reported	58	58 *Rhinovirus*	93 Not reported	RT-PCR for respiratory specimens (viruses) ^c^	58 Not reported	58 Not reported	58 Not reported	(NOS, 6) 57 survived 1 died
Le Roux et al. 2020 [76], France	Retrospective case report, single-center	1	1	10	1 (100) AND 1 White (Caucasian)	0	0	2	1 *Varicella zoster* virus 1 *Rotavirus*	1 Amoxicillin/clavulanic acid 1 Azithromycin 1 Acyclovir	PCR	0	0	0	(Modified NOS, high) 1 survived
Leclercq et al. 2021 [77], Switzerland	Retrospective case report, single-center	1	1	96	1 (100) AND 1 White (Caucasian)	1	0	1	1 EBV 1 Group A *Streptococcus*	1 Amoxicillin 1 Cephalosporin	RT-PCR for respiratory specimens (viruses) ^c^ Serum antibody tests (IgM, IgG)	0	0	0	(Modified NOS, high) 1 survived
Lee et al. 2022 [78], United States	Retrospective cohort, single-center	1625	92	Not reported	Gender (not reported) AND Ethnicity (not reported)	0	0	111	56 RSV 38 *Influenza* A virus 11 *Rhinovirus* 2 *Influenza* B virus 2 *Adenovirus* 2 *Parainfluenza* virus	Not reported	RT-PCR for respiratory specimens (viruses) ^c^ Serum antibody tests (IgM, IgG)	Not reported	Not reported	Not reported	(NOS, 7) Treatment outcome (not reported)
Leuzinger et al. 2020 [79], Switzerland	Retrospective cohort, single-center	16	4	Age group ≤60: 2 (14.3%) patients (viral coinfection) Age group ≤192: 2 (14.3%) patients (viral coinfection)	Gender (not reported) AND 4 White (Caucasian)	0	0	8	4 *Rhinovirus* 2 RSV 2 *Parainfluenza* virus (types 1–4)	4 Not reported	RT-PCR for respiratory specimens (viruses) ^c^	4 Not reported	4 Not reported	4 Not reported	(NOS, 7) Treatment outcome (not reported)
Li et al. 2020 [80], China	Retrospective cohort, single-center	40	15	Mean ± SD, 61 ± 56	Gender (not reported) AND 15 Asian	14	0	4	13 *Mycoplasma pneumoniae* 3 *Influenza* A or B virus 1 *Adenovirus* 1 *Streptococcus pneumonia*	13 Azithromycin 1 Meropenem 1 Piperacillin/tazobactam	RT-PCR for respiratory specimens (viruses) ^c^	1	1	1	(NOS, 7) 15 survived
Li et al. 2021 [81], China	Retrospective cohort, single-center	81	27	Mean ± SD, 76.5 ± 9.6	15 (55.6) AND 27 Asian	24	0	6	20 *Mycoplasma pneumoniae* 1 *Influenza* A virus 2 *Influenza* B virus 1 RSV 1 *Adenovirus* 1 *Parainfluenza* virus 2 3 *Moraxella catarrhalis* 1 *Streptococcus pneumoniae*	27 Not reported	RT-PCR for respiratory specimens (viruses) ^c^ Sputum (bacteria) Serum antibody tests (IgM, IgG)	1	1	1	(NOS, 7) 27 survived
Lin et al. 2020 [82], China	Retrospective cohort, single-center	92	1	36	0 (0) AND 1 Asian	0	0	1	1 *Metapneumovirus*	1 Not reported	RT-PCR for respiratory specimens (viruses) ^c^	1 Not reported	1 Not reported	1 Not reported	(Modified NOS, high) Treatment outcome (not reported)
Ma et al. 2020 [83], China	Retrospective cohort, single-center	45	4	4 Not reported	Gender (not reported) AND 4 Asian	0	0	7	4 *Mycoplasma pneumonia* 2 *Parainfluenza* virus 1 *Adenovirus*	4 Not reported	RT-PCR for respiratory specimens (viruses) ^c^	3	3	3	(NOS, 6) Treatment outcome (not reported)
Mania et al. 2022 [84], Poland	Retrospective cohort, multicenter	1283	135	Median (IQR), 72 (12–156)	Gender (not reported) AND 135 White (Caucasian)	15	0	37	11 *Streptococcus pneumoniae* 2 *Influenza* A virus 2 *Escherichia coli* 1 *Adenovirus* 1 *Rhinovirus* 1 *Bocavirus* 1 RSV 1 *Parainfluenza* 1 *Mycoplasma pneumoniae* 1 *Klebsiella oxytoca* 2 *Varicella zoster* virus 3 *Herpes simplex* virus 25 *Rotavirus*, *adenovirus*, and *norovirus*	135 Not reported	RT-PCR for respiratory specimens (viruses) ^c^ Blood, urine, and pharyngeal swabs (culture)	3	0	2	(NOS, 7) 135 survived
Mannheim et al. 2020 [85], United States	Retrospective case series, single-center	10	4	Median (IQR), 132 (84–192)	Gender (not reported) AND Ethnicity (not reported)	2	0	4	1 *Mycoplasma pneumoniae* 2 *Adenovirus* 1 *Rhinovirus* 1 *Escherichia coli* 1 *Rotavirus*	4 Not reported	RT-PCR for respiratory specimens (viruses) ^c^ Serum antibody test (IgM) Urine (culture)	4	0	0	(NOS, 7) 4 survived
Mansour et al. 2020 [86], Lebanon	Retrospective case report, single-center	1	1	16	0 (0) AND 1 Arab	1	0	0	1 *Streptococcus pneumoniae*	1 Ceftriaxone 1 Metronidazole	Blood (culture)	0	0	0	(Modified NOS, high) 1 survived
Marsico et al. 2022 [87], Italy	Retrospective case report, single-center	1	1	<1	0 (0) AND 1 White (Caucasian)	1	0	0	1 Multidrug-resistant *Enterobacter asburiae*	1 Azithromycin 1 Vancomycin 1 Ceftazidime 1 Gentamycin 1 Meropenem 1 Aztreonam 1 Ceftazidime/avibactam 1 Fosfomycin	Blood (culture)	1	1	1	(Modified NOS, high) 1 survived
Mathur et al. 2022 [11], India	Retrospective cohort, single-center	327	17	Mean (SD), 137 (32)	9 (52.9) AND 17 Indian	17	0	0	17 *Mycobacterium tuberculosis*	17 Not reported	Blood culture (bacteria)	6	2	7	(NOS, 7) 13 survived 4 died
Mithal et al. 2020 [88], United States	Retrospective case series, single-center	18	2	<3	1 (50) AND 2 Hispanic	2	0	2	2 RSV 1 *Streptococcus agalactiae* 1 *Klebsiella oxytoca*	1 Antibiotics	RT-PCR for respiratory specimens (viruses) ^c^ Urine (culture)	0	0	0	(Modified NOS, high) 2 survived
Mohammadi et al. 2022 [89], Iran	Retrospective cohort, single-center	45	4	1, 36, 72, and 120	2 (50) AND 4 Persian	0	0	4	4 *Adenovirus*	0	RT-PCR for respiratory specimens (viruses) ^c^	0	0	0	(NOS, 5) 4 survived
Moin et al. 2021 [90], Pakistan	Retrospective cohort, single-center	4238	4	≤ 180 (10–180)	4 (100) AND 4 Pakistani	0	4	0	1 *Candida auris* 1 *Candida albicans* 1 *Candida tropicalis* 1 *Candida rugosa*	4 Antibiotics 4 Antifungals	Blood (culture)	1	1	1	(NOS, 7) 3 survived 1 died
Morand et al. 2020 [91], France	Retrospective case report, single-center	1	1	55	0 (0) AND 1 White (Caucasian)	0	0	1	1 EBV	0	RT-PCR for respiratory specimens (viruses) ^c^	0	0	0	(Modified NOS, high) 1 survived
Mulale et al. 2021 [19], Botswana	Retrospective case report, single-center	1	1	3	1 (100) AND 1 Black	1	0	0	1 Rifampin-sensitive *Mycobacterium tuberculosis*	1 Ampicillin 1 Gentamicin 1 Rifampicin 1 Isoniazid 1 Pyrazinamide 1 Ethambutol	PCR assay for gastric lavage (bacteria)	1	1	1	(Modified NOS, high) 1 died
Ng et al. 2020 [92], United Kingdom	Retrospective case series, single-center	8	3	12, 0.5, and 10	1 (33.3) AND 3 White (Caucasian)	0	0	5	2 *Adenovirus* 2 *Rhinovirus* 1 Other coronaviruses (229E, NL63, and OC43)	1 Amoxicillin 1 Cefotaxime 1 Gentamicin	RT-PCR for respiratory specimens (viruses) ^c^	1	0	0	(Modified NOS, high) 3 survived
Nieto-Moro et al. 2020 [93], Spain	Retrospective case report, single-center	1	1	8	1 (100) AND 1 White (Caucasian)	1	0	0	1 *Streptococcus pneumoniae*	1 Azithromycin 1 Clindamycin 1 Meropenem 1 Linezolid	Blood (culture)	1	0	1	(Modified NOS, high) 1 survived
Nygaard et al. 2022 [20], Denmark	Retrospective case series, multicenter	2	2	24 and 132	1 (50) AND 2 White (Caucasian)	2	0	2	2 Panton-Valentine leukocidin-producing *Staphylococcus aureus* 1 *Parainfluenza* 1 *Rhinovirus*	1 Meropenem 1 Clindamycin 1 Amoxicillin	Blood PCR assays (viruses) Blood, lung biopsy and CSF (culture)	1	1	1	(Modified NOS, high) 2 died
Oba et al. 2020 [94], Brazil	Retrospective case report, single-center	1	1	2	0 (0) AND 1 Hispanic	1	0	0	1 *Clostridium difficile*	0	Fecal PCR assays (bacteria)	1	0	0	(Modified NOS, high) 1 survived
Ogunbayo et al. 2022 [95], South Africa	Retrospective cohort, multicenter	36	31	Median (IQR), 16 (5–29)	19 (61.3) AND 31 Black	0	0	53	23 *Rhinovirus* 16 RSV 6 *Adenovirus* 8 *Parainfluenza* virus 3	31 Not reported	RT-PCR for respiratory specimens (viruses) ^c^	2	31 Not reported	31 Not reported	(NOS, 7) Treatment outcome (not reported)
Palmero et al. 2020 [96], Argentina	Retrospective case series, multicenter	4	4	Range (60–192)	Gender (not reported) AND 4 Hispanic	4	0	0	4 *Mycobacterium tuberculosis*	4 Isoniazid 4 Rifampicin 4 Pyrazinamide 4 Ethionamide	Blood culture (bacteria)	1	1	1	(Modified NOS, high) 3 survived 1 died
Patek et al. 2020 [97], United States	Retrospective case report, single-center	1	1	0.5	1 (100) AND 1 White (Caucasian)	1	0	0	1 MSSA	1 Antibiotic 1 Acyclovir	Wound (culture)	1	0	1	(Modified NOS, high) 1 survived
Peng et al. 2020 [98], China	Retrospective cohort, single-center	75	42	Mean ± SD, 72.7 ± 57.4	Gender (not reported) AND 42 Asian	31	0	8	28 *Mycoplasma pneumoniae* 1 *Moraxella catarrhalis* 1 *Staphylococcus aureus* 1 *Streptococcus pneumoniae* 3 *Influenza* B virus 1 *Influenza* A virus 2 *Adenoviridae* 1 *Cytomegalovirus* 1 RSV	37 1st- or 2nd-generation cephalosporins 28 Azithromycin	RT-PCR for respiratory specimens (viruses) ^c^ Serum antibody test (IgM) for *Mycoplasma pneumoniae* (only)	0	0	1	(NOS, 7) 42 survived
Pigny et al. 2021 [99], Switzerland	Retrospective cohort, single-center	51	7	Median (IQR), 50.4 (20.4–87.6)	Gender (not reported) AND 7 White (Caucasian)	0	0	9	4 *Rhinovirus* 2 Other coronaviruses (NL63) 2 *Adenovirus* 1 *Metapneumovirus*	7 Not reported	RT-PCR for respiratory specimens (viruses) ^c^	7 Not reported	7 Not reported	7 Not reported	(NOS, 6) Treatment outcome (not reported)
Plebani et al. 2020 [100], Italy	Retrospective case series, single-center	9	4	36, 120, 168, and 120	2 (50) AND 4 White (Caucasian)	4	0	0	4 *Mycoplasma pneumonia*	3 Ceftriaxone 1 Cefotaxime 2 Azithromycin 1 Ampicilline/sulbactam 1 Clindamycin	Serum antibody test (IgM)	4 Not reported	4 Not reported	4 Not reported	(Modified NOS, high) 4 survived
Pokorska-Śpiewak et al. 2021 [101], Poland	Prospective case series, single-center	15	1	1 Not reported	Gender (not reported) AND 1 White (Caucasian)	0	0	1	1 *Influenza* A virus	0	RT-PCR for respiratory specimens (viruses) ^c^	0	0	0	(Modified NOS, high) 1 survived
Pucarelli-Lebreiro et al. 2022 [102], Brazil	Prospective cohort, single-center	105	9	Median, 45	Gender (not reported) AND 9 Hispanic	0	0	10	6 RSV 1 *Influenza* 2 *Rhinovirus* 1 *Norovirus*	9 Not reported	RT-PCR for respiratory specimens (viruses) ^c^	0	0	0	(NOS, 7) 9 survived
Rastogi et al. 2022 [103], India	Retrospective case series, single-center	19	1	108	0 (0) AND 1 Indian	1	0	0	1 *Mycobacterium tuberculosis*	1 Isoniazid 1 Rifampicin 1 Pyrazinamide 1 Ethionamide	PCR assay of bronchoalveolar lavage (bacteria)	0	0	0	(NOS, 7) 1 survived
Ratageri et al. 2021 [104], India	Retrospective case report, single-center	1	1	96	1 (100) AND1 Indian	0	0	1	1 *Dengue* virus	0	IgM antibody test (dengue)	0	0	0	(Modified NOS, high) 1 survived
Raychaudhuri et al. 2021 [105], India	Prospective cohort, single-center	102	43	Median (IQR), 54 (4.8–90)	23 (53.4) AND 43 Indian	26	0	12	4 MRSA 5 MSSA 3 CONS 3 *Pseudomonas aeruginosa* 1 *Klebsiella pneumonia* 7 *Scrub typhus* 5 *Dengue* 3 *Salmonella typhi* 1 *Hepatitis* A 1 EBV 2 RSV 1 *Influenza* A virus 1 *Adenovirus* 1 *Rhinovirus*	38 Antibiotics	RT-PCR for respiratory specimens (viruses) ^c^ Blood, respiratory secretions, and CSF (culture)	27	15	14	(NOS, 8) 39 survived 4 died
Rebelo et al. 2022 [21], Portugal	Retrospective case report, single-center	1	1	168	1 (100) AND 1 White (Caucasian)	1	0	0	1 *Neisseria meningitidis* serogroup B	1 Ceftriaxone 1 Meropenem 1 Vancomycin	Blood (culture)	1	1	1	(Modified NOS, high) 1 died
Said et al. 2022 [106], Saudi Arabia	Retrospective case report, single-center	1	1	10	Gender (not reported) AND 1 Arab	1	0	0	1 *Escherichia coli*	1 Antibiotic	Urine (culture)	0	0	0	(NOS, 6) 1 survived
Sanchez Solano and Sharma 2022 [107], United States	Retrospective case report, single-center	1	1	192	1 (100) AND 1 White (Caucasian)	1	0	0	1 MRSA	1 Ceftriaxone 1 Vancomycin 1 Clindamycin	Bronchoalveolar lavage (culture)	1	1	1	(Modified NOS, high) 1 survived
Santoso et al. 2021 [108], Indonesia	Retrospective cohort, multicenter	90	1	1 Not reported	Gender (not reported) AND 1 Asian	0	0	1	1 *Dengue* virus	1 Not reported	*Dengue* NS1 antigen IgM and IgG antibody tests (*dengue*)	1 Not reported	1 Not reported	1 Not reported	(NOS, 7) Treatment outcome (not reported)
Schober et al. 2022 [109], Multi-country	Retrospective cohort, multicenter	403	54	45.4 (6.4–129.2)	Gender (not reported) AND Ethnicity (not reported)	24	0	32	24 Bacterial 32 Viral	3 Azithromycin	RT-PCR for respiratory specimens (viruses) ^c^ Blood (culture)	10	4	4	(NOS, 7) Treatment outcome (not reported)
See et al. 2020 [110], Malaysia	Retrospective case reports, multicenter	4	1	48	0 (0) AND 1 Asian	0	0	1	1 *Influenza* A virus	1 Phenoxymethylpenicillin	RT-PCR for respiratory specimens (viruses)c	0	0	0	(Modified NOS, high) 1 survived
Serrano et al. 2020 [111], Spain	Retrospective case report, single-center	1	1	96	1 (100) AND 1 White (Caucasian)	1	0	0	1 *Mycoplasma pneumonia*	1 Not reported	IgM and IgG antibody tests (*Mycoplasma pneumonia*)	0	0	0	(Modified NOS, high) 1 survived
Shabrawishi et al. 2021 [112], Saudi Arabia	Retrospective case series, single-center	7	1	168	0 (0) AND 1 Arab	1	0	0	1 *Mycobacterium tuberculosis*	1 Ceftriaxone 1 Azithromycin 1 Isoniazid 1 Rifampicin 1 Pyrazinamide 1 Ethionamide	Blood culture (bacteria)	0	0	0	(Modified NOS, high) 1 survived
Shi et al. 2020 [113], China	Retrospective case report, single-center	1	1	3	1 (100) AND 1 Asian	0	0	1	1 RSV	1 Ceftizoxime	RT-PCR for respiratory specimens (viruses) ^c^	1	0	1	(Modified NOS, high) 1 survived
Sibulo et al. 2021 [114], United States	Retrospective case report, single-center	1	1	36	1 (100) AND 1 White (Caucasian)	1	0	0	1 *Staphylococcus epidermidis*	1 Vancomycin 1 Clindamycin 1 Piperacillin/tazobactam	Blood (culture)	1	1	0	(Modified NOS, high) 1 survived
Şık et al. 2022 [115], Turkey	Retrospective cohort, single-center	14	1	3	1 (100) AND 1 White (Caucasian)	0	0	1	1 *Rhinovirus*		RT-PCR for respiratory specimens (viruses) ^c^	0	0	1	(NOS, 7) 1 survived
Somasetia et al. 2020 [22], Indonesia	Retrospective case report, single-center	1	1	72	1 (100) AND 1 Asian	0	0	1	1 *Dengue* virus	1 Antibiotics	IgM antibody test (*dengue*)	1	1	1	(Modified NOS, high) 1 died
Sun et al. 2020 [116], China	Retrospective cohort, single-center	36	23	Mean (range), 6.43 (2–12)	Gender (not reported) AND 23 Asian	23 Not reported	23 Not reported	23 Not reported	Unspecified number of *Cytomegalovirus*, EBV and *Mycoplasma pneumonia*	15 Cefmetazole 15 Azithromycin	RT-PCR for respiratory specimens (viruses) ^c^	1	1	1	(NOS, 7) 22 survived 1 died
Sun et al. 2020 [117], China	Retrospective case series, single-center	8	1	96	1 (100) AND 1 Asian	0	0	1	1 *Influenza* A virus	1 Antibiotics	RT-PCR for respiratory specimens (viruses) ^c^	1	1	1	(Modified NOS, high) 1 Remained in ICU
Tadolini et al. 2020 [118], Multi-country	Retrospective cohort, multicenter	49	1	3	1 (100) AND 1 Black	1	0	0	1 *Mycobacterium tuberculosis*	1 Antibiotics 1 Isoniazid 1 Rifampicin 1 Pyrazinamide 1 Ethionamide	Blood culture (bacteria)	0	0	0	(NOS, 7) 1 survived
Tagarro et al. 2021 [119], Spain	Retrospective cohort, multicenter	41	2	Median (IQR), 36 (10.8–72)	Gender (not reported) AND Ethnicity (Not reported)	0	0	2	2 *Influenza* B virus	2 Not reported	RT-PCR for respiratory specimens (viruses) ^c^	0	0	0	(NOS, 7) 2 survived
Tan et al. 2020 [120], China	Retrospective case series, single-center	10	3	24, 105, and 111	1 (33.3) AND 3 Asian	4	0	0	3 *Mycoplasma pneumonia* 1 *Chlamydia pneumonia*	1 Antibiotics	Serum antibody test (IgM)	3 Not reported	3 Not reported	3 Not reported	(Modified NOS, high) Treatment outcome (not reported)
Taweevisit et al. 2022 [23], Thailand	Retrospective case report, single-center	1	1	67	1 (100) AND 1 Asian	2	1	4	1 *Aspergillus* species 1 *Cytomegalovirus* 1 *Pseudomonas aeruginosa* 1 *Acinetobacter baumannii* 1 *Adenovirus* 1 EBV 1 *Herpes* virus 4	1 Antibiotics	Alveolar fluid (culture) RT-PCR for respiratory specimens (viruses) ^c^ Serum antibody tests (IgM and IgG)	1	1	1	(Modified NOS, high) 1 died
Tchidjou et al. 2021 [121], France	Retrospective case report, single-center	1	1	1.5	1 (100) AND 1 White (Caucasian)	1	0	0	1 *Citrobacter koseri*	1 Cefotaxime 1 Gentamycin 1 Amoxicillin/clavulanic acid	Urine (culture)	0	0	0	(Modified NOS, high) 1 survived
Tiwari et al. 2020 [122], India	Retrospective case report, single-center	1	1	168	0 (0) AND 1 Indian	0	0	1	1 *Dengue* virus	1 Ceftriaxone 1 Azithromycin	*Dengue* NS1 antigen IgM antibody test (*dengue*)	1	0	1	(Modified NOS, high) 1 survived
Trifonova et al. 2022 [123], Bulgaria	Retrospective cohort, multicenter	242	16	All patients were <192 156 (*n* = 1) 36 (*n* = 1)	Gender (not reported) AND 16 White (Caucasian)	16 Not reported	16 Not reported	2	2 *Influenza* A virus	16 Not reported	RT-PCR for respiratory specimens (viruses) ^c^	1	0	0	(NOS, 7) 16 survived
Vanzetti et al. 2020 [124], Argentina	Retrospective, case reports, single-center	1	1	204	1 (100) AND 1 Hispanic	1	0	0	1 *Mycobacterium tuberculosis*	1 Isoniazid 1 Rifampicin 1 Pyrazinamide 1 Ethionamide	PCR assay (bacteria) Sputum (culture)	0	0	0	(Modified NOS, moderate) 1 survived
Varela et al. 2022 [125], Brazil	Prospective cohort, multicenter	92	31	Median (IQR), 64.8 (24–122.4)	Gender (not reported) AND 31 Hispanic	0	0	30	29 *Rhinovirus* 1 *Enterovirus*	5 Azithromycin	RT-PCR for respiratory specimens (viruses) ^c^	4	0	0	(NOS, 7) 31 survived
Verheijen et al. 2022 [126], The Netherlands	Retrospective case report, single-center	1	1	0.03	0 (0) AND 1 White (Caucasian)	1	0	0	1 *Staphylococcus aureus*	1 Flucloxacillin	RT-PCR for respiratory specimens (viruses) ^c^ Blood (culture)	1	1	1	(Modified NOS, high) 1 survived
Vidal et al. 2022 [127], Multi-country	Retrospective cohort, multicenter	29	12	Median, 36	Gender (not reported) AND 12 White (Caucasian)	0	0	12	12 *Adenovirus*	12 Not reported	RT-PCR for respiratory specimens (viruses) ^c^	2	12 Not reported	12 Not reported	(NOS, 7) Treatment outcome (not reported)
Vu et al. 2021 [128], United States	Retrospective case report, single-center	1	1	48	1 (100) AND 1 White (Caucasian)	1	0	0	1 *Streptococcus pneumonia*	1 Cefepime 1 Vancomycin 1 Ceftriaxone 1 Amoxicillin	Pleural fluid (culture)	1	1	1	(Modified NOS, high) 1 survived
Wanga et al. 2021 [129], United States	Retrospective cohort, multicenter	713	113	Age group <12: 37 (32.4%) patients (viral coinfection) Age group 12–48: 41 (36.1%) patients (viral coinfection)	Gender (not reported) AND Ethnicity (not reported)	113 Not reported	113 Not reported	113	113 RSV	113 Not reported	RT-PCR for respiratory specimens (viruses) ^c^	113 Not reported	113 Not reported	113 Not reported	(NOS, 6) Treatment outcome (not reported)
Wehl et al. 2020 [130], Germany	Retrospective case report, single-center	1	1	4	Gender (not reported) AND 1 White (Caucasian)	0	0	1	1 *Influenza* A virus	0	RT-PCR for respiratory specimens (viruses) ^c^	0	0	0	(Modified NOS, high) 1 survived
Wu et al. 2020 [131], China	Retrospective cohort, multicenter	34	19	72 (1.2–180.9)	Gender (not reported) AND 19 Asian	16	0	10	16 *Mycoplasma pneumoniae* 3 RSV 3 EBV 3 *Cytomegalovirus* 1 *Influenza* A and B virus	15 Azithromycin	RT-PCR for respiratory specimens (viruses) ^c^	1	0	1	(NOS, 7) 19 survived
Xia et al. 2020 [132], China	Retrospective case series, single-center	20	8	Median, 24	Gender (not reported) AND 8 Asian	4	0	5	1 *Cytomegalovirus* 2 *Influenza* B virus 1 *Influenza* A virus 4 *Mycoplasma pneumoniae* 1 RSV	8 Not reported	RT-PCR for respiratory specimens (viruses) ^c^	0	0	0	(Modified NOS, high) 8 survived
Yakovlev et al. 2022 [133], Russia	Retrospective cohort, single-center	287	32	Median (IQR), 12 (8.4–30) (viral coinfection) Median (IQR), 144 (90–180) (bacterial coinfection)	Gender (not reported) AND 32 White (Caucasian)	16	0	34	11 *Rhinovirus* 11 Other coronaviruses (HKU-1/OC 43) 9 *Mycoplasma pneumoniae* 7 *Chlamydia pneumoniae* 4 *Metapneumovirus* 4 *Parainfluenza* virus 3 4 *Parainfluenza* virus 4	32 Not reported	RT-PCR for respiratory specimens (viruses) ^c^ Serum antibody tests (IgM and IgG)	6	32 Not reported	32 Not reported	(NOS, 7) Treatment outcome (not reported)
Zeng et al. 2020 [134], China	Retrospective cohort, single-center	3	1	7.75	1 (100) AND 1 Asian	1	0	0	1 *Enterobacter*	1 Antibiotics	Blood (culture)	1	1	1	(NOS, 7) 1 survived
Zhang et al. 2020 [135], China	Retrospective case series, multicenter	34	16	Median (IQR), 33 (10–94.2)	Gender (not reported) AND 16 Asian	9	0	15	9 *Mycoplasma pneumoniae* 6 *Influenza* B virus 3 *Influenza* A virus 2 RSV 2 EBV 1 *Parainfluenza* virus 1 *Adenovirus*	11 Antibiotics 9 Azithromycin	RT-PCR for respiratory specimens (viruses) ^c^	0	0	0	(Modified NOS, high) 16 survived
Zhang et al. 2021 [136], United States	Retrospective case series, multicenter	16	2	Mean ± SD, 204 ± 61.3	Gender (not reported) AND Ethnicity (not reported)	0	0	4	1 *Rhinovirus* 1 *Adenovirus* 1 RSV 1 *Influenza* A virus	2 Antibiotics	RT-PCR for respiratory specimens (viruses) ^c^	2 Not reported	2 Not reported	2 Not reported	(Modified NOS, high) Treatment outcome (not reported)
Zheng et al. 2020 [137], China	Retrospective cohort, multicenter	25	3	Median (IQR), 36 (24–108)	2 (66.7) AND 3 Asian	4	0	2	3 *Mycoplasma pneumoniae* 2 *Influenza* B virus 1 *Enterobacter aerogenes*	1 Meropenem 1 Linezolid	RT-PCR for respiratory specimens (viruses) ^c^	1	1	1	(NOS, 7) 3 survived
Zheng et al. 2020 [138], China	Retrospective cohort, single-center	4	1	180	1 (100) AND 1 Asian	0	0	1	1 *Influenza* B virus	1 Antibiotics	RT-PCR for respiratory specimens (viruses) ^c^	0	0	0	(NOS, 7) 1 survived
Zhu et al. 2020 [139], China	Retrospective cohort, single-center	257	11	<180	Gender (not reported) AND 11 Asian	20	2	3	6 *Streptococcus pneumoniae* 5 *Haemophilus influenzae* 3 *Klebsiella pneumoniae* 3 *Staphylococcus aureus* 2 *Aspergillus* 1 *Metapneumovirus* 1 *Cytomegalovirus* 1 *Mycoplasma pneumonia* 1 *Adenovirus* 1 *Pseudomonas aeruginosa* 1 *Escherichia coli*	11 Not reported	RT-PCR for respiratory specimens (viruses) ^c^	0	0	0	(NOS, 7) 11 survived
Zou et al. 2020 [140], China	Retrospective case report, single-center	2	2	28 and 156	1 (50) AND 2 Asian	0	0	2	2 *Influenza* A virus	1 Cefaclor	Serum antibody tests (IgM and IgG)	0	0	0	(Modified NOS, high) 2 survived

Abbreviations: ARDS, acute respiratory distress syndrome; CONS, coagulase-negative *Staphylococcus* species; CMV, *Cytomegalovirus*; COVID-19, coronavirus disease 2019; CSF, cerebrospinal fluid; EBV, *Epstein–Barr virus*; ICU, intensive care unit; IgG, immunoglobulin G; IgM, immunoglobulin M; IQR, interquartile range; MRSA, Methicillin-resistant *Staphylococcus aureus*; MSSA, Methicillin-susceptible *Staphylococcus aureus*; NOS, Newcastle–Ottawa scale; RT-PCR, real-time reverse transcription–polymerase chain reaction; RSV, respiratory syncytial virus; SARS-CoV-2, severe acute respiratory syndrome coronavirus 2; SD, standard deviation. ^a^ Data are presented as median (25th–75th percentiles), or mean ± SD. ^b^ Patients of black ethnicity include African-American, Black African, African, and Afro-Caribbean patients. ^c^ PCR assay for multiple respiratory viruses (including *influenza virus* types A and B, *respiratory syncytial virus* type A/B, *human metapneumovirus*, *parainfluenza virus* types 1–4, other coronaviruses (229E, NL63, and OC43), *metapneumovirus*, *rhinovirus*, *enterovirus*, *adenovirus*, *parechovirus*, and *bocavirus*).

### 3.2. Demographic, Clinical Characteristics, and Treatment Outcomes of Children with COVID-19 and Bacterial, Fungal, and/or Respiratory Viral Coinfection

The included studies comprised a total of 17,588 children with confirmed SARS-CoV-2 infection who were tested for co-pathogens, as detailed in Table 1. Among these 17,588 COVID-19 patients, bacterial, fungal, and/or respiratory viral coinfections were reported (*n* = 1633, 9.3%). The median patient age ranged from 1.4 months to 144 months across studies. There was an increased male predominance in pediatric COVID-19 patients diagnosed with bacterial, fungal, and/or viral coinfections in most of the studies (male gender: *n* = 204, 59.1% compared to female gender: *n* = 141, 40.9%) [6,8,10,11,16,17,18,19,21,22,23,31,35,38,40,45,46,47,48,52,54,57,59,60,61,63,64,67,68,69,70,71,74,76,77,81,90,93,95,97,104,105,107,111,113,114,117,118,121,124,128,134,137,138]. The majority of the cases belonged to White (Caucasian) (*n* = 441, 53.3%) [6,7,9,13,20,21,36,38,39,41,42,44,47,48,50,52,58,60,62,63,65,69,71,75,76,77,79,84,87,91,93,97,99,100,101,107,111,114,115,121,123,126,127,128,130,133], Asian (*n* = 205, 24.8%) [8,9,22,23,37,45,54,65,66,68,80,81,82,83,98,108,110,113,116,117,120,131,132,134,135,137,138,139,140], Indian (*n* = 71, 8.6%) [11,31,51,63,72,74,103,104,105,122], and Black (*n* = 51, 6.2%) [6,9,15,19,46,55,59,60,63,95,118] ethnicities.

COVID-19 children coinfected with bacteria, fungi, and/or respiratory viruses were reported to have received antibiotics in 77 studies [5,8,9,12,13,14,15,16,19,20,21,22,23,36,38,39,40,42,45,46,50,51,52,55,56,59,60,63,64,65,66,67,68,69,70,72,74,75,76,77,80,86,87,88,90,92,93,96,97,98,100,103,105,106,107,109,110,112,113,114,116,117,118,120,121,122,124,125,126,128,131,134,135,136,137,138,140]. The most prescribed antibiotics were azithromycin (*n* = 109) [9,15,36,39,64,65,70,76,80,87,93,98,100,109,112,116,122,125,131,135], 1st/2nd/3rd generation of cephalosporins (*n* = 66) [12,13,42,45,60,65,67,77,87,98,100,113,116,121,128,140], ceftriaxone (*n* = 29) [12,14,21,38,39,51,52,60,67,68,72,74,86,100,107,112,122,128], isoniazid (*n* = 13) [19,51,55,59,74,96,103,112,118,124], pyrazinamide (*n* = 13) [19,51,55,59,74,96,103,112,118,124], rifampicin (*n* = 13) [19,51,55,59,74,96,103,112,118,124], ethionamide (*n* = 12) [51,55,59,74,96,103,112,118,124], meropenem (*n* = 11) [16,20,21,40,50,74,75,80,87,93,137], vancomycin (*n* = 11) [13,16,21,60,72,74,87,107,114,128], amoxicillin/clavulanic acid (*n* = 9) [9,45,59,60,76,121], amoxicillin (*n* = 8) [14,15,20,40,67,77,92,128], clindamycin (*n* = 8) [13,20,38,60,67,93,100,107,114], ampicillin/sulbactam (*n* = 7) [39,60,100], and gentamycin (*n* = 6) [9,19,42,87,92,121]. There were children who were admitted to the intensive care unit (*n* = 214, 18.6%) [4,5,6,7,8,9,11,12,13,15,16,18,19,20,21,22,23,35,36,39,42,46,51,53,56,58,61,63,64,66,67,72,74,80,81,83,84,85,87,90,92,93,94,95,96,97,105,107,109,113,114,116,117,122,123,125,126,127,128,131,133,134,137], intubated and placed on mechanical ventilation (*n* = 98, 9.2%) [4,5,6,7,8,9,11,12,13,15,16,17,18,19,20,21,22,23,35,36,39,42,46,51,56,58,61,67,72,74,80,81,83,87,90,96,105,107,109,114,116,117,126,128,134,137], and suffered acute respiratory distress syndrome (*n* = 100, 12.5%) [4,6,7,8,9,11,12,13,16,17,18,19,20,21,22,23,39,42,45,46,48,51,55,56,58,61,66,67,72,74,80,81,83,84,87,90,93,96,97,98,105,107,109,113,115,116,117,122,126,128,131,134,137].

Clinical treatment outcomes for the COVID-19 children who were coinfected with bacteria, fungi, and/or respiratory viruses and died was documented in 43 (4.4%) cases [4,5,6,7,8,9,10,11,12,13,14,15,16,17,18,19,20,21,22,23,90,96,105,116], while 931 (95.6%) of the COVID-19 cases recovered [4,5,6,7,8,9,10,11,31,33,34,35,36,38,40,42,43,45,46,47,48,50,51,52,54,55,56,57,59,60,62,63,64,65,66,67,68,69,70,72,74,75,76,77,80,81,84,85,86,87,88,89,90,91,92,93,94,95,96,97,100,101,102,103,104,105,106,107,110,111,112,113,114,115,116,118,119,121,122,123,124,125,126,128,130,131,132,134,135,137,138,139,140], and final treatment outcome was reported in one patient who remained in the intensive care unit (*n* = 1, %) [117].

### 3.3. Meta-Analysis of Bacterial, Fungal, and Respiratory Viral Coinfections in Children with SARS-CoV-2

**Figure 3:**
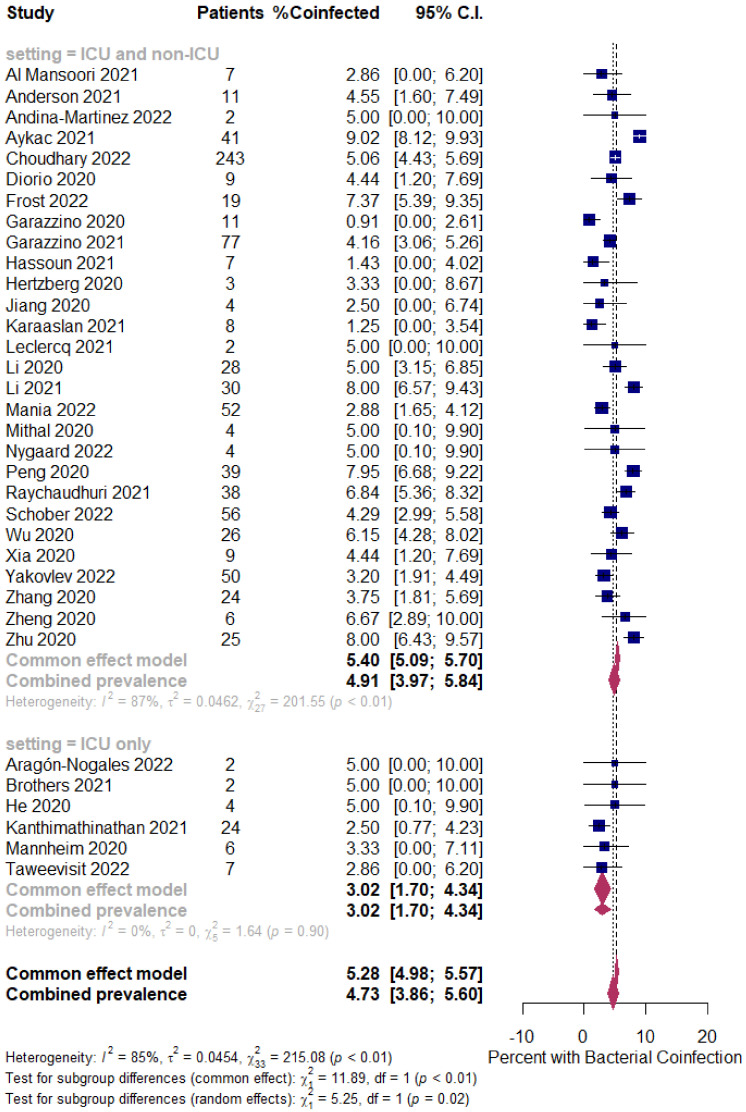
Pooled estimate for the prevalence of bacterial coinfections in children with COVID-19 stratified by the ICU admission (ICU and non-ICU compared to ICU only). [4,5,6,7,8,9,12,13,20,23,32,36,39,57,58,63,64,66,69,77,80,81,84,85,88,98,105,109,131,132,133,135,137,139].

**Figure 4:**
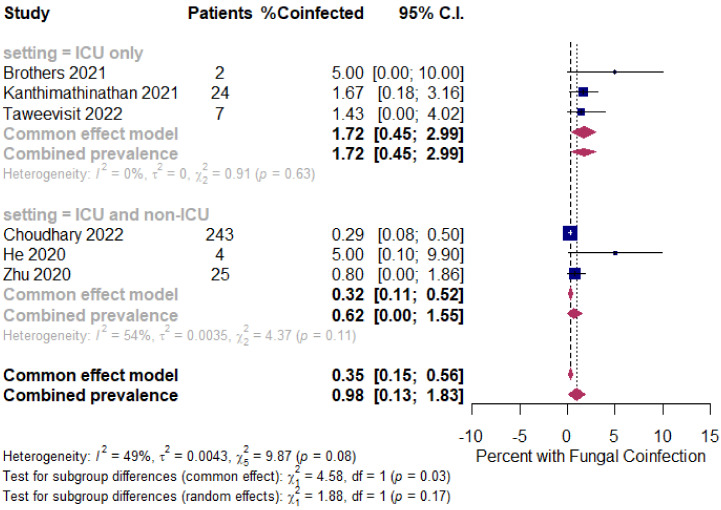
Pooled estimate for the prevalence of fungal coinfections in children with COVID-19 stratified by the ICU admission (ICU and non-ICU compared to ICU only). [5,8,9,13,23,139].

**Figure 5:**
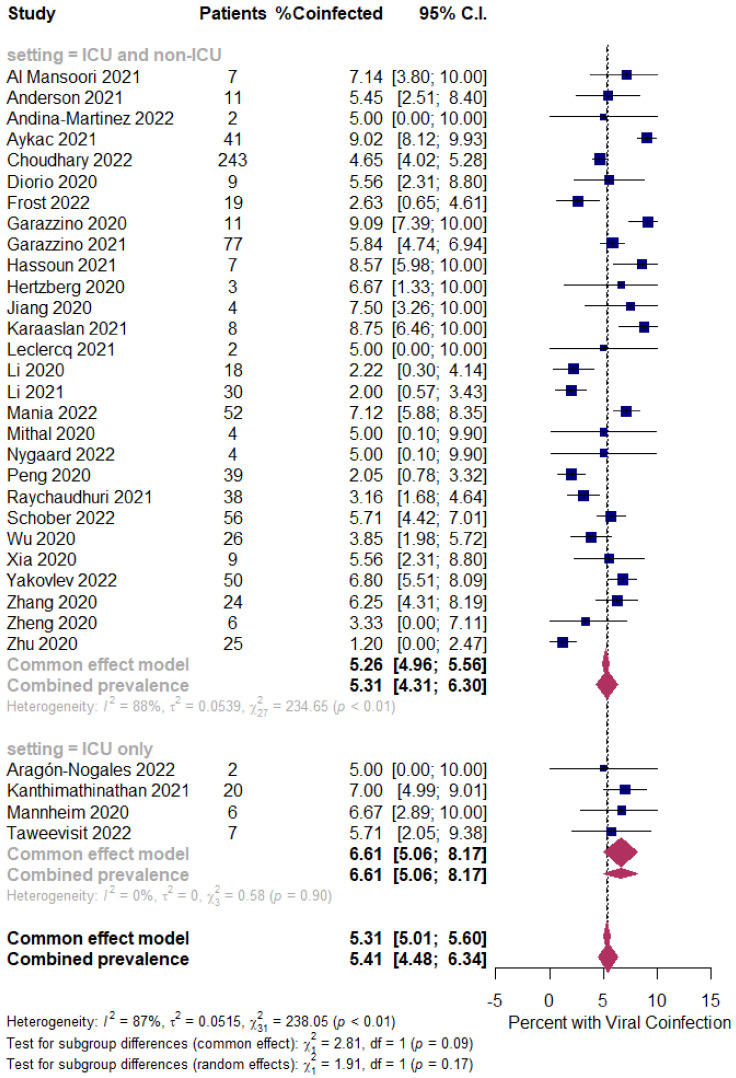
Pooled estimate for the prevalence of respiratory viral coinfections in children with COVID-19 stratified by the ICU admission (ICU and non-ICU compared to ICU only). [4,5,6,7,9,12,20,23,32,36,39,57,58,63,64,66,69,77,80,81,84,85,88,98,105,109,131,132,133,135,137,139].

The overall pooled proportions of COVID-19 children who had laboratory-confirmed bacterial, fungal, and respiratory viral coinfections were 4.73% (95% CI 3.86 to 5.60, *n* = 445, 34 studies, *I*^2^ 85%, *p* < 0.01), 0.98% (95% CI 0.13 to 1.83, *n* = 17, six studies, *I*^2^ 49%, *p* < 0.08), and 5.41% (95% CI 4.48 to 6.34, *n* = 441, 32 studies, *I*^2^ 87%, *p* < 0.01), respectively; (Figure 3, Figure 4 and Figure 5).

In bacterial coinfected COVID-19 children, subgroup analysis showed some difference in the rates between all patients (patients in the ICU and non-ICU group or ICU only group); the ICU and non-ICU group showed a prevalence of 4.91% (95% CI 3.97 to 5.84, *n* = 431, 28 studies, *I*^2^ 87%, *p* < 0.01), while the ICU only group showed a prevalence of 3.02% (95% CI 1.70 to 4.34, *n* = 14, six studies, *I*^2^ 0%, *p* = 0.90), respectively; Figure 3.

In fungal coinfected COVID-19 children, subgroup analysis showed almost a threefold increase in the rates between all patients (patients in the ICU and non-ICU group or ICU only group); the ICU only group showed a prevalence of 1.72% (95% CI 0.45 to 2.99, *n* = 11, three studies, *I*^2^ 0%, *p* = 0.63), while the ICU and non-ICU group showed a prevalence of 0.62% (95% CI 0.00 to 1.55, *n* = 6, three studies, *I*^2^ 54%, *p* = 0.11), respectively; Figure 4.

However, in the respiratory viral coinfected COVID-19 children, subgroup analysis showed a slight difference in the rates between all patients (patients in the ICU and non-ICU group or ICU only group); the ICU and non-ICU group showed a prevalence of 5.31% (95% CI 4.31 to 6.30, *n* = 418, 28 studies, *I*^2^ 88%, *p* < 0.01), while the ICU only group showed a prevalence of 6.61% (95% CI 5.06 to 8.17, *n* = 23, four studies, *I*^2^ 0%, *p* = 0.90), respectively; Figure 5.

**Figure 6:**
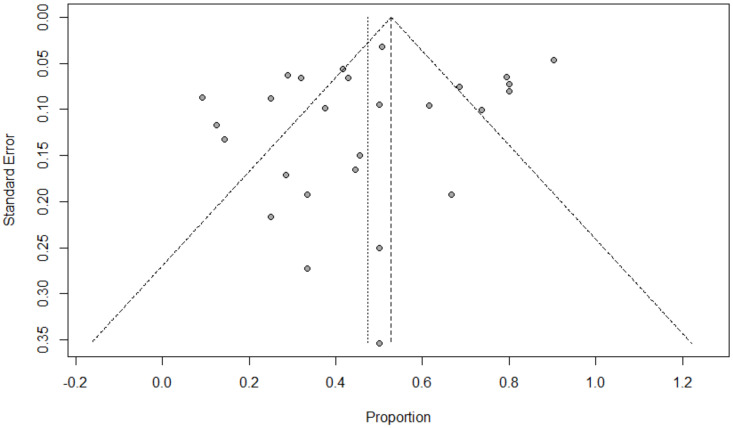
Funnel plot to evaluate publication bias for the pooled effect size to estimate the prevalence of bacterial coinfections in children with COVID-19 based on ICU admission.

**Figure 7:**
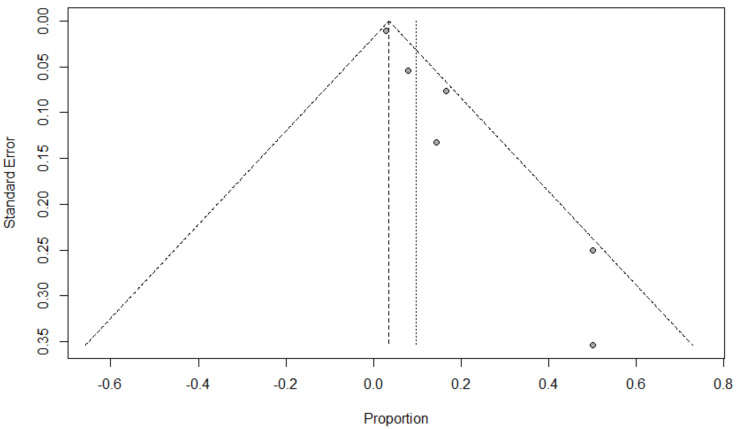
Funnel plot to evaluate publication bias for the pooled effect size to estimate the prevalence of fungal coinfections in children with COVID-19 based on ICU admission.

**Figure 8:**
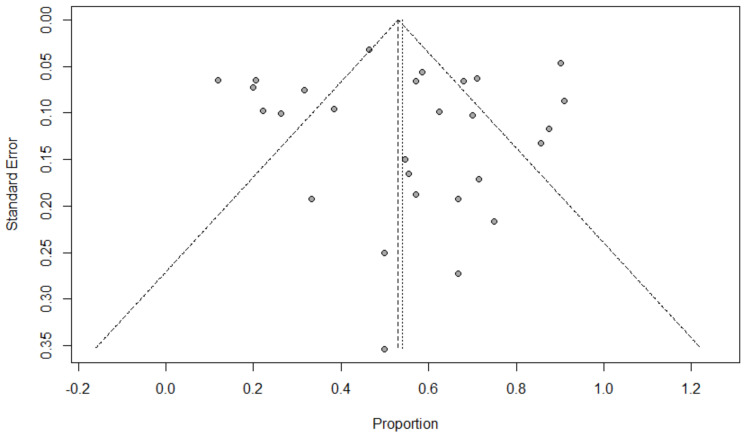
Funnel plot to evaluate publication bias for the pooled effect size to estimate the prevalence of respiratory viral coinfections in children with COVID-19 based on ICU admission.

Funnel plots for possible publication bias for the pooled effect size to determine the prevalence of bacterial, fungal, and/or fungal coinfections in children with COVID-19 appeared asymmetrical on visual inspection, and Egger’s tests confirmed asymmetry with *p*-values < 0.05; Figure 6, Figure 7 and Figure 8.

### 3.4. Bacterial, Fungal, and Respiratory Viral Co-Pathogens in COVID-19 Children

Specific bacterial co-pathogens were reported in 71/130 (54.6%) studies, which is about 31.8% of the reported coinfections. The most common bacteria were *Mycoplasma pneumoniae* (*n* = 120), *Streptococcus pneumoniae* (*n* = 65), *Mycobacterium tuberculosis* (*n* = 31), *Staphylococcus aureus* (*n* = 12), *Escherichia coli* (*n* = 11), *Haemophilus influenza* (*n* = 10), *Chlamydia pneumoniae* (*n* = 9), and *Pseudomonas aeruginosa* (*n* = 9) (Table 2).

Fungal co-pathogens were reported in 8/130 (6.1%) studies, which is equal to only 1.4% of the reported coinfections. The most common fungal organisms were *Aspergillus* species (*n* = 3), fungal bezoars (*n* = 2), *Candida albicans* (*n* = 1), *Candida auris* (*n* = 1), *Candida glabrata* (*n* = 1), *Candida rugosa* (*n* = 1), and *Candida tropicalis* (*n* = 1) (Table 3). 

Respiratory viral co-pathogens were reported in 88/130 (67.7%) studies, representing about 66.8% of the reported coinfections. The most common respiratory viruses were RSV (*n* = 342), Rhinovirus (*n* = 209), *Influenza* A virus (*n* = 80), *Adenovirus* (*n* = 60), *Parainfluenza* virus (types 1–4) (*n* = 29), *Influenza* B virus (*n* = 28), *Metapneumovirus* (*n* = 27), EBV (*n* = 14), *Cytomegalovirus* (*n* = 12), *Dengue* virus (*n* = 12), Coronaviruses (HKU-1/OC 43) (*n* = 11), and *Bocavirus* (*n* = 10) (Table 4).

## 4. Discussion

This systematic review and meta-analysis included 17,588 laboratory-confirmed COVID-19 children from 130 observational studies to estimate the prevalence of coinfections with bacteria, fungi, and/or respiratory viruses. Children with SARS-CoV-2 infection had the following prevalence of pathogen coinfections: bacterial (4.7%, 95% CI 3.8–5.6), fungal (0.9%, 95% CI 0.1–1.8), and respiratory viral (5.4%, 95% CI 4.4–6.3). COVID-19 children had higher fungal and respiratory viral coinfections in ICU units (1.7%, 95% CI 0.4–2.9 and 6.6%, 95% CI 5–8.1, respectively) than mixed ICU and non-ICU patients. However, bacterial coinfection was lower in children infected with SARS-CoV-2 in ICU group (3%, 95% CI 1.7–4.3). Children with COVID-19 seem to have a distinctly lower susceptibility to bacterial, fungal, and/or respiratory viral coinfections than adults. Our study documents that 4.7% (bacteria), 0.9% (fungal), and 5.4% (viral) of the pediatric COVID-19 population harbor microbiologically confirmed coinfections, which is much lower than the recent systematic review and meta-analysis, including 72 studies, conducted from 1 December 2019 to 31 March 2021, portraying coinfection rates of 15.9% (bacterial), 3.7% (fungal), and 6.6% (viral) in the adult COVID-19 population [141]. Lower rates of bacterial, fungal, and/or respiratory viral coinfection in children with SARS-CoV-2 infection compared to the adult COVID-19 population may have different explanations. Immunologically, children seem to have an immature receptor system, immune-system-specific regulatory mechanisms, and possible cross-protection from other common bacterial, fungal, and viral infections occurring in children [142,143]. A growing body of evidence suggests that children’s immune systems can neutralize SARS-CoV-2 because their T cells are relatively naïve and mostly untrained, and thus might have a greater capacity to respond to new viruses and eliminate SARS-CoV-2 before it replicates in large numbers [144,145,146]. Children are also the main reservoir for seasonal coronaviruses, and some researchers have suggested that antibodies for these coronaviruses might confer some protection against SARS-CoV-2 [143,146]. Moreover, children are more protected at the cellular level, as the expression of angiotensin-converting enzyme 2, which is the receptor that SARS-CoV-2 uses for host entry, is less frequently expressed in the epithelial cells of the nasal passages and lungs of younger children [147]. Otherwise, differences can be explained by the numerous different study designs to a large extent, as well as selection bias, consideration of respiratory and extra-respiratory pathogens, microbiological investigations employed, use of culture and non-culture methods, time of specimen collection, exclusion/inclusion of contaminants, climate, temporal variations in microbial epidemiology and the study population itself. 

Three previous systematic reviews and meta-analyses reported on bacterial, fungal, and respiratory vial coinfections; however, these studies included mixed populations of adults and children, included a smaller number of studies (with most data for adults and very few pediatric patients), and sensitivity analysis to study the proportion of coinfection in COVID-19 children was not conducted [148,149,150]. To the best of our knowledge, this is the first and largest systematic review and meta-analysis to report exclusively on bacterial, fungal, and respiratory viral coinfection in children with COVID-19, and we pooled evidence from 130 studies, including at least *Mycoplasma pneumoniae*, *Streptococcus pneumoniae*, *Mycobacterium tuberculosis*, *Staphylococcus aureus*, RSV, *rhinovirus*, *influenza A* or *B* virus, *adenovirus*, *parainfluenza* virus, and *metapneumovirus* due to their virulence and prevalence, in an attempt to avoid measurement bias. Of the 98.6% who had additional respiratory viruses or bacteria detected, we found that the most common identified virus and bacterium in children with COVID-19 were RSV (*n* = 342, 31.4%) and *Mycoplasma pneumonia* (*n* = 120, 23.1%), in line with findings in two previous systematic reviews and meta-analyses, which reported that RSV and *Mycoplasma pneumonia* were the most commonly isolated co-pathogens in the adult population with SARS-CoV-2 infection [148,150]. RSV and *Mycoplasma pneumonia* cause acute respiratory tract illness in people of all ages, and all children are infected with RSV by 2 years of age [151], while approximately one-half of patients infected with *Mycoplasma pneumonia* are <6 years old [school-age years) [152]. RSV is the most common cause of lower respiratory tract infection in children <1 year of age [153], and bronchiolitis (up to 80% of which is caused by RSV) is a leading cause of hospital admission [154] and an important cause of death in infants and young children [155]. *Mycoplasma pneumonia* is the second most common cause of respiratory tract infections, and upper and lower respiratory tracts may be affected [156]. This pathogen causes a wide spectrum of illness, ranging from asymptomatic to severe community-acquired pneumonia or extrapulmonary manifestations necessitating ICU admission [157,158]. Several countries have reported that there has been a suppression of RSV and *Mycoplasma pneumonia* circulation, and their typical seasonality, since early 2020 due to the preventive infection control measures and non-pharmaceutical interventions against SARS-CoV-2 [159,160,161,162,163,164]. However, RSV and *Mycoplasma pneumonia* activity rebounded in early–mid 2021 at a fast pace, as public health restrictions and social distancing regulations were relaxed; higher hospitalization rates were reported, and most of the hospitalized children required ICU admission [165,166,167]. Although two recent studies demonstrated no association between SARS-CoV-2 and RSV coinfection and clinical severity (need or use of supplemental oxygen, ICU admission, mechanical ventilation, and mortality), the evidence was only based on three small studies [167,168]. In contrast, evidence of clinical severity regarding cases coinfected with SARS-CoV-2 and *Mycoplasma pneumonia* is well-established, and several studies reported such coinfection as being associated with an increase in inpatient mortality, length of hospital stay, and need for mechanical ventilation [69,100,169,170]. In children, both RSV and *Mycoplasma pneumonia* are similar to SARS-CoV-2; as potential triggers for a cytokine storm, leading to the development of Multisystem Inflammatory Syndrome in Children (MIS-C), they appear to play a role in the pathogenesis, and may contribute to the subsequent clinical severity of COVID-19. The cytokines tumor necrosis factor-alpha, interleukin-8, interleukin-6, and interleukin-1 beta were detected in the airway secretions of children infected with RSV and *Mycoplasma pneumonia*, which may act as a double whammy of respiratory pathogens and correlate with severe pathogenesis [171,172,173,174]. As coinfection with either the highly contagious RSV or *Mycoplasma pneumonia* and SARS-CoV-2 can modify the disease course and contribute to severity, and can cause serious compilations in children, especially those with high-risk comorbidities, healthcare workers need to consider RSV or *Mycoplasma pneumonia* and SARS-CoV-2 coinfection in the differential diagnosis of acute febrile illness in the endemic areas.

It is noteworthy that in the studies where the laboratory techniques for co-pathogen detection were described, a high number of bacterial and viral coinfections in children infected with SARS-CoV-2 included in our review were diagnosed serologically through the detection of immunoglobulins M and/or G. One of the easiest, most convenient, and fastest point-of-care testing to diagnose COVID-19 and other bacterial, fungal, and/or respiratory co-pathogens is by rapid serology tests; however, serology testing has been associated with many false-positive antibody test results for COVID-19 and mixed pathogens [111,175,176]. Therefore, application of serologic laboratory techniques for co-pathogen detection across all studies was likely to reveal an even higher overall coinfection proportion and high rates of anti-infective use for admitted children with SARS-CoV-2 infection to treat documented or presumed bacterial, fungal, and/or respiratory viral coinfections [177,178,179]. In line with previous studies, we identified high anti-infective use in pediatric patients with COVID-19 [177,180,181]. As the prevalence of bacterial, fungal, or respiratory viral coinfections in children with COVID-19 is not high, and anti-infectives likely provide minimal benefit as an empirical treatment, clinicians should prescribe anti-infectives wisely, and only in cases with an objective diagnosis of coinfection, as injudicious use of anti-infectives is associated with unintended consequences, such as adverse events, toxicity, resistance, *Clostridioides difficile* infections, risk of emergence and transmission of multidrug-resistant organisms, morbidity, and death [182,183,184,185,186,187]. Undoubtedly, coinfection in children with COVID-19 is likely to be an important modifier in the development of these abovementioned unintended consequences; however, the degree to which co-pathogens interact with SARS-CoV-2 remains unclear in many cases, and even where we know that interactions are occurring, the mechanisms are often poorly defined [188,189].

The combined pooled prevalence for fungal coinfections reported in our review in COVID-19 children is very low (0.98%). In general, very low numbers of fungal species, out of thousands of fungi, are pathogenic [190], and fungal infections in children, other than those caused by *Candida* species, are uncommon [191]. This can be explained by the strong natural immunity towards fungi in healthy children, and almost every invasive fungal infection that occurs in children is opportunistic [192]. In line with previous studies, all children infected with SARS-CoV-2 who were coinfected with fungi had recognized risk factors for fungaemia, such as use of central lines, malignancy, renal failure, mechanical ventilation, immunosuppression, neutropenia, solid organ transplant recipients, and use of broad-spectrum parenteral antibiotics and corticosteroids [193,194]. Fungal infections in children can be curbed by early diagnosis and timely treatment with the optimal prescription of antifungals based on culture and susceptibility tests, along with adopting appropriate hygienic and sanitization measures [195,196].

### Limitations of the Study

We acknowledge that our study is not without some limitations. First, while all of the evidence discussed was based on many cohorts and case series, and some case reports, many of these were small and performed in single centers, and not necessarily generalizable to children infected with SARS-CoV-2 who had bacterial, fungal, or respiratory viral coinfections. Second, almost all studies included in this review were retrospective in design, except seven prospective studies, which could have introduced potential reporting bias due to reliance on obtaining illness histories regarding the identified pediatric cases with COVID-19 and coinfection from household members or contacts and clinical case records. Third, to asses factors associated with the clinical severity in children infected with SARS-CoV-2 who have coinfections, a larger cohort of patients is needed. Last, the study was not registered in Prospero, an international prospective register of systematic reviews, as this might have added extra work and the merit was mostly limited to the avoidance of duplication.

## 5. Conclusions

Children with COVID-19 seem to have distinctly lower rates of bacterial, fungal, and/or respiratory viral coinfections than adults. RSV and *Mycoplasma pneumonia* were the most common identified virus and bacterium in children infected with SARS-CoV-2. Knowledge of bacterial, fungal, and/or respiratory viral confections has potential diagnostic and treatment implications in COVID-19 children.

## Figures and Tables

**Table 2:** Proportion of all identified bacterial co-pathogens in children with COVID-19 (N = 520).

Bacterial Pathogen Type	Identified Number (%)
Unspecified bacteria	181 (34.8)
*Mycoplasma pneumoniae*	120 (23.1)
*Streptococcus pneumoniae*	65 (12.5)
*Mycobacterium tuberculosis*	31 (6)
*Staphylococcus aureus*	12 (2.3)
*Escherichia coli*	11 (2.1)
*Haemophilus influenza*	10 (1.9)
*Chlamydia pneumoniae*	9 (1.7)
*Pseudomonas aeruginosa*	9 (1.7)
MSSA	8 (1.5)
*Moraxella catarrhalis*	7 (1.3)
*Scrub typhus*	7 (1.3)
MRSA	6 (1.1)
*Salmonella typhi*	5 (1)
Group A *Streptococcus*	4 (0.8)
*Klebsiella pneumoniae*	4 (0.8)
CONS	3 (0.6)
*Acinetobacter baumannii*	2 (0.4)
*Bordetella pertussis*	2 (0.4)
*Klebsiella oxytoca*	2 (0.4)
*Klebsiella pneumoniae*	2 (0.4)
*Neisseria meningitidis*	2 (0.4)
*Prevotella species*	2 (0.4)
*Streptococcus constellatus*	2 (0.4)
*Streptococcus agalactiae*	1 (0.2)
*Streptococcus intermedius*	1 (0.2)
*Streptococcus mitis*	1 (0.2)
*Citrobacter koseri*	1 (0.2)
*Clostridium difficile*	1 (0.2)
*Enterobacter*	1 (0.2)
*Enterobacter aerogenes*	1 (0.2)
*Enterobacter cloacae*	1 (0.2)
*Enterobacter asburiae*	1 (0.2)
*Escherichia hermannii*	1 (0.2)
Gram-negative *bacilli*	1 (0.2)
*Mycobacterium bovis*	1 (0.2)
*Salmonella enteritis*	1 (0.2)
*Staphylococcus epidermidis*	1 (0.2)

Abbreviations: CONS, coagulase-negative *Staphylococcus* species; COVID-19, coronavirus disease 2019; MRSA, Methicillin-resistant *Staphylococcus aureus*; MSSA, Methicillin-susceptible *Staphylococcus aureus.*

**Table 3:** Proportion of all identified fungal co-pathogens in children with COVID-19 (N = 23).

Fungal Pathogen Type	Identified Number (%)
Unspecified fungi	13 (56.5)
*Aspergillus* species	3 (13)
Fungal bezoars	2 (8.7)
*Candida albicans*	1 (4.3)
*Candida auris*	1 (4.3)
*Candida glabrata*	1 (4.3)
*Candida rugosa*	1 (4.3)
*Candida tropicalis*	1 (4.3)

**Table 4:** Proportion of all identified respiratory viral co-pathogens in children with COVID-19 (N = 1090).

Viral Pathogen Type	Identified Number (%)
RSV	342 (31.4)
*Rhinovirus*	209 (19.2)
Unspecified viruses	198 (18.2)
*Influenza A* virus	80 (7.3)
*Adenovirus*	60 (5.5)
*Parainfluenza* virus (types 1–4)	29 (2.7)
*Influenza B* virus	28 (2.6)
*Metapneumovirus*	27 (2.5)
*Rotavirus*, *adenovirus*, and *norovirus*	25 (2.3)
EBV	14 (1.3)
*Cytomegalovirus*	12 (1.1)
*Dengue* virus	12 (1.1)
Coronaviruses (HKU-1/OC 43)	11 (1)
*Bocavirus*	10 (0.9)
Coronaviruses (229E, NL63, and OC43)	6 (0.5)
*Enterovirus*	5 (0.4)
*Herpes simplex* virus	5 (0.4)
Coronavirus NL63	5 (0.4)
*Varicella zoster* virus	4 (0.4)
*Rotavirus*	2 (0.2)
*Human Herpesvirus 6*	1 (0.1)
*Norovirus*	1 (0.1)
*Parechovirus*	1 (0.1)
*Parvovirus B19*	1 (0.1)
*Hepatitis A* virus	1 (0.1)
*Herpes virus 4*	1 (0.1)

Abbreviations: EBV, Epstein–Barr virus; RSV, respiratory syncytial virus.

## Data Availability

Not applicable.

## Abbreviations

ARDS, acute respiratory distress syndrome; CMV, *Cytomegalovirus*; CONS, coagulase-negative *Staphylococcus* species; COVID-19, coronavirus disease 2019; CSF, cerebrospinal fluid; EBV, *Epstein–Barr virus*; ICU, intensive care unit; IgG, immunoglobulin G; IgM, immunoglobulin M; MRSA, Methicillin-resistant *Staphylococcus aureus*; MSSA, Methicillin-susceptible *Staphylococcus aureus*; NOS, Newcastle–Ottawa scale; PRISMA, Preferred Reporting Items for Systematic Reviews and Meta-Analyses; RSV, respiratory syncytial virus; RT-PCR, real-time reverse transcription–polymerase chain reaction; SARS-CoV-2, severe acute respiratory syndrome coronavirus 2.
